# Tpr Deficiency Disrupts Erythroid Maturation With Impaired Chromatin Condensation in Zebrafish Embryogenesis

**DOI:** 10.3389/fcell.2021.709923

**Published:** 2021-10-13

**Authors:** Shuang Wu, Kai Chen, Tao Xu, Ke Ma, Lei Gao, Cong Fu, Wenjuan Zhang, Changbin Jing, Chunguang Ren, Min Deng, Yi Chen, Yi Zhou, Weijun Pan, Xiaoe Jia

**Affiliations:** ^1^Shanghai Institute of Nutrition and Health, Chinese Academy of Sciences, Shanghai, China; ^2^Central Laboratory, Qingdao Agricultural University, Qingdao, China; ^3^State Key Laboratory for Medical Genomics, Shanghai Institute of Hematology, Ruijin Hospital, Shanghai Jiao Tong University School of Medicine, Shanghai, China; ^4^Stem Cell Program, Hematology/Oncology Program at Children’s Hospital Boston, Harvard Medical School, Boston, MA, United States; ^5^Inner Mongolia Key Laboratory of Hypoxic Translational Medicine, Baotou Medical College, Baotou, China

**Keywords:** erythrocytes, erythroid maturation, chromatin condensation, zebrafish, Tpr

## Abstract

Vertebrate erythropoiesis involves nuclear and chromatin condensation at the early stages of terminal differentiation, which is a unique process to distinguish mature erythrocytes from erythroblasts. However, the underlying mechanisms of chromatin condensation during erythrocyte maturation remain elusive. Here, we reported a novel zebrafish mutant^*cas*7^ with erythroid maturation deficiency. Positional cloning showed that a single base mutation in *tprb* gene, which encodes nucleoporin translocated promoter region (Tpr), is responsible for the disrupted erythroid maturation and upregulation of erythroid genes, including *ae1-globin* and *be1-globin*. Further investigation revealed that deficient erythropoiesis in *tprb*^*cas7*^ mutant was independent on HIF signaling pathway. The proportion of euchromatin was significantly increased, whereas the percentage of heterochromatin was markedly decreased in *tprb*^*cas7*^ mutant. In addition, *TPR* knockdown in human K562 cells also disrupted erythroid differentiation and dramatically elevated the expression of globin genes, which suggests that the functions of TPR in erythropoiesis are highly conserved in vertebrates. Taken together, this study revealed that Tpr played vital roles in chromatin condensation and gene regulation during erythroid maturation in vertebrates.

## Introduction

The zebrafish (*Danio rerio*) is a well-established model organism to study hematopoietic development due to its unique advantages (Jong and Zon, 2005; Paik and Zon, 2010). Similar to mammalian hematopoiesis, zebrafish hematopoiesis occurs in two distinct waves. The first primitive wave of hematopoiesis occurs in two different regions: the posterior lateral mesoderm that forms the intermediate cell mass later and the anterior lateral mesoderm, which generate primitive erythroid cells and myeloid cells, respectively (Chen and Zon, 2009; Paik and Zon, 2010). With the start of the heartbeat at 24 h post-fertilization (hpf), these primitive blood cells enter circulation throughout the embryo. The second definitive wave of hematopoiesis is characterized by the formation of self-renewing hematopoietic stem cells (HSCs) in the aorta–gonad–mesonephros region (Bertrand et al., 2010; Kissa and Herbomel, 2010). Then the nascent HSCs migrate to the caudal hematopoietic tissue and colonize in kidney marrow finally (Murayama et al., 2006; Li et al., 2018).

Almost in all vertebrates, erythropoiesis is a stepwise process that involves differentiation of HSCs to committed burst forming unit-erythroid (BFU-E) followed by colony forming unit-erythroid (CFU-E) (Dzierzak and Philipsen, 2013; Palis, 2014). In terminal erythropoiesis, CFU-Es differentiate to mature erythrocytes and can be divided into four morphologically distinguishable cell types: proerythroblasts, basophilic erythroblasts, polychromatophilic erythroblasts, and orthochromatophilic erythroblasts (Schwartz, 2016). Also, many progressive characteristics are involved in terminal erythropoiesis including several cell divisions, decrease in cell size, nuclear and chromatin condensation, and extrusion of mitochondria and even the nucleus (Palis, 2014).

Although nuclear chromatin condensation is a common feature to define morphologically distinctive erythroblasts at different developmental stages, enucleation is unique to mammals, and nuclei are still retained in mature circulating erythrocytes in fish and avian (Ji et al., 2011). Chromatin condensation is also required for terminal erythropoiesis, as defects in this process are associated with anemia and myelodysplastic syndrome (Wickramasinghe and Wood, 2005; Menon and Ghaffari, 2021). Previous studies have identified several architectural factors including linker histone H5 and nuclear serpin MENT that promote avian erythroid chromatin condensation (Verreault and Thomas, 1993; Istomina et al., 2003). At the same time, histone deacetylation and caspase-3 are necessary for mammalian chromatin condensation (Zermati et al., 2001; Popova et al., 2009; Zhao et al., 2016). However, the detailed mechanisms of chromatin condensation are unclear.

Nucleoporin Tpr (translocated promoter region) is an architectural component of nuclear pore complex (NPC), which locates on the nucleoplasmic side of the pore to form a nuclear basket structure (Cordes et al., 1997; Krull et al., 2004). It exists as a homodimer *via* the N-terminal coiled-coil domain to be one of the eight basket filaments, whereas the C-terminal domain appears unfolded and flexible into the nucleus (Hase et al., 2001). Tpr has been implicated in a variety of nuclear functions, including chromatin organization and transcriptional regulation, nuclear transport, and mitosis. Megator (*Drosophila* Tpr) and its binding partner Nup153 have been shown to bind to 25% of the genome in continuous domains that are transcriptionally active (Vaquerizas et al., 2010). Also, Tpr was shown to be required for heterochromatin exclusion zones (HEZs) at nuclear pores after poliovirus infection (Krull et al., 2010) and maintain an open chromatin state favorable for HIV replication (Lelek et al., 2015). Recently, Tpr has been reported to be necessary for formation and maintenance of senescence-associated heterochromatin foci (SAHFs) during oncogenic Ras-induced senescence (Boumendil et al., 2019). However, Tpr-mediated chromatin organization during developmental processes *in vivo* remains uncertain.

In this study, we reported an essential role of Tpr in terminal erythropoiesis by characterizing a zebrafish mutant^*cas*7^ with a missense mutation in *tprb* gene. The homozygous *tprb* mutant manifested erythroid cell maturation blockage and abnormal erythroid gene expression. Tpr deficiency did not induce the activation of HIF signaling pathway, and *hif-2a* knockdown could not rescue abnormal erythroid phenotypes in *tprb*^*cas7*^ mutant. The elevated erythroid gene expression including *ae1-globin* and *be1-globin* in *tprb*^*cas7*^ mutants was due to their enhanced transcriptional activity, rather than the increased number of erythrocytes. Further investigation revealed that nuclear chromatin condensation and organization were strikingly disrupted specifically in erythrocytes in *tprb*^*cas7*^ mutant, but not in other tissue cells. In addition, in human K562 cells, *TPR* knockdown also caused blocking of erythroid differentiation and a dramatic increase in α globin and β globin gene expression, indicating that the function of TPR in erythropoiesis is highly conserved in mammalian. Our findings not only revealed the critical role of Tpr in chromatin condensation and gene regulation but also highlighted the importance of nuclear pore proteins in erythroid maturation.

## Results

### Erythroid Maturation Is Disrupted in Zebrafish Mutant^*cas7*^

**FIGURE 1:**
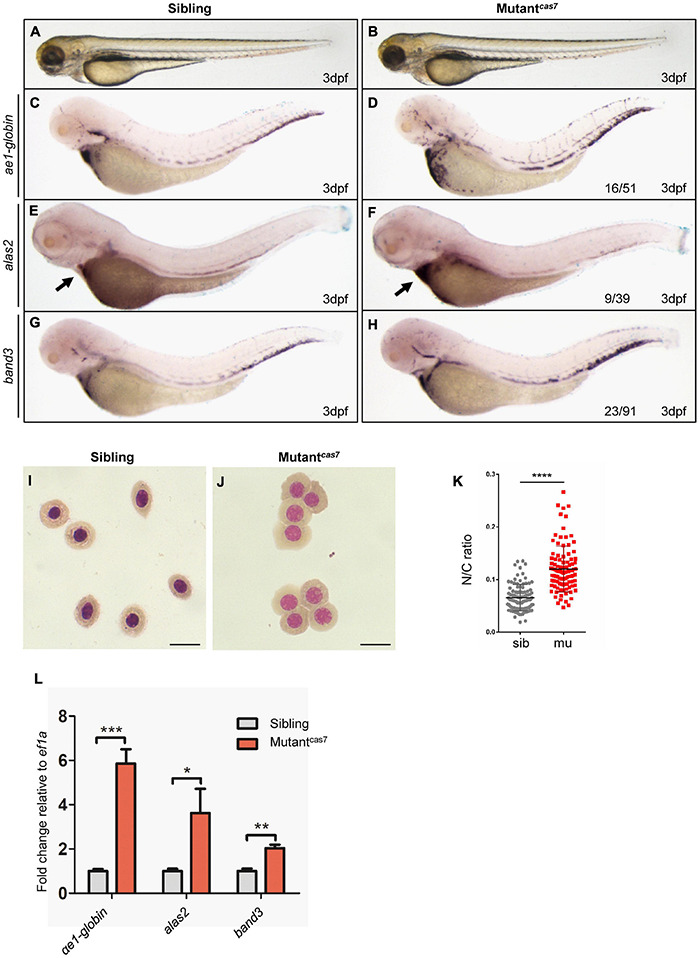
Erythroid maturation is disrupted in zebrafish mutant^*cas*7^. **(A,B)** Light microscope images of zebrafish wild-type (WT) sibling and mutant^*cas*7^ embryos at 3 dpf. **(C–H)** WISH analysis of *ae1-globin* **(C,D)**, *alas2* **(E,F)**, and *band3* **(G,H)** expression in sibling and mutant^*cas*7^ embryos at 3 dpf. After WISH and photographing, all embryos were extracted for genomic DNA and genotyped by sequencing, then the mutant percentage was evaluated. The number of embryos in a het-het incross clutch with the expression pattern is shown in the mutant^*cas*7^ column and corresponding percentages are listed inside each panel. Arrow in panel **(E,F)** indicates the *alas2* staining in heart region. **(I,J)** Giemsa staining for erythrocytes at 4 dpf in sibling and mutant^*cas*7^. Relative to the number of orthochromatophilic erythroblasts, mutant^*cas*7^ displayed a marked increase in circulating polychromatophilic erythroblasts compared with sibling. Scale bars represent 10 μm. **(K)** Statistical analysis of the nucleus-to-cytoplasm (N/C) ratio in sibling and mutant^*cas*7^. *N* = 100, error bars represent SEM. ****p ≤ 0.0001. **(L)** The relative expression of α*e1-globin*, *alas2*, *band3* in sibling and mutant^*cas*7^ embryos at 3 dpf. Error bars represent SEM. *p ≤ 0.05; **p ≤ 0.01; *** *p* ≤ 0.001.

In a large-scale ENU mutagenesis screen for erythroid development mutations, we obtained *cas7*, a novel mutant with erythrocyte development defects and recessive lethality. The mutant^*cas*7^ embryos were morphologically indistinguishable from wild-type siblings before 3 dpf, with normal blood flow and heart beats, and survived to about 6 dpf (Figures 1A,B). Whole-mount *in situ* hybridization (WISH) revealed that the expressions of erythroid markers (*ae1-globin*, *alas2*, and *band3*) were all increased abnormally (Figures 1C–H). Consistent with WISH analysis, real-time qPCR also showed the same result (Figure 1L). During the time course of α*e1-globin* WISH analysis, we did not detect any difference between sibling and mutant^*cas*7^ before 3 dpf (Supplementary Figures 1A–D). The marginal decrease of α*e1-globin* expression was detectable until 3 dpf (Supplementary Figures 1E,F), while the mutant^*cas*7^ embryos were morphologically distinguishable from wild-type siblings after 4 dpf.

Erythroid maturation is a stepwise process typically including dramatically decrease in cell size, and the N/C (nucleus-to-cytoplasm) ratio is a robust parameter for terminal differentiation, which had been reported previously (Fraser et al., 2007). Therefore, we can use the N/C ratio as a quantitative marker for erythroid maturation. Comparison of circulating erythrocytes from sibling and mutant^*cas*7^ embryos showed impaired maturation in the latter, for the latter contains more polychromatophilic erythroblasts while the former was abundant in orthochromatophilic erythroblasts at 4 dpf (Figures 1I,J; Qian et al., 2007). Also, the N/C ratio was higher in mutant^*cas*7^, confirming the impaired erythrocyte maturation (Figure 1K).

To examine other hematopoietic lineages, we firstly performed WISH analysis of definitive hematopoietic cells markers including *cmyb* (hematopoietic stem and progenitor cell marker), *mpx* (granulocyte marker), *lyz* (macrophage marker), and *rag-1* (lymphocyte marker). The expression of these markers was identical between sibling and mutant^*cas*7^ at 3 or 4 dpf, which indicated that the definitive hematopoiesis was normal (Supplementary Figures 2A–H). Consistent with WISH results, the real-time qPCR suggested that the expressions of these definitive hematopoietic cell markers (*cmyb, mpo, lyz*, and *rag-1*) were unchanged (Supplementary Figure 2I). However, the expression levels of erythroid genes were not all increased, such as *sdhb*, *fth1a* (iron-related protein gene), and *hebp2* (heme binding protein gene) (Supplementary Figure 2I). Then, we investigated the primitive hematopoiesis and vascular morphogenesis in mutant^*cas*7^. WISH analysis showed that the expression of *scl* (hematopoietic progenitor marker), *gata1* (erythrocyte progenitor marker), *pu.1* (myeloid progenitor marker), *mpx*, and *kdrl* (endothelial cell marker) were also normal in mutant^*cas*7^ at 22 or 26 hpf (Supplementary Figure 3). Taken together, these results suggested that erythroid maturation was specifically disrupted in zebrafish mutant^*cas*7^.

### Positional Cloning in Zebrafish Mutant^*cas7*^

**FIGURE 2:**
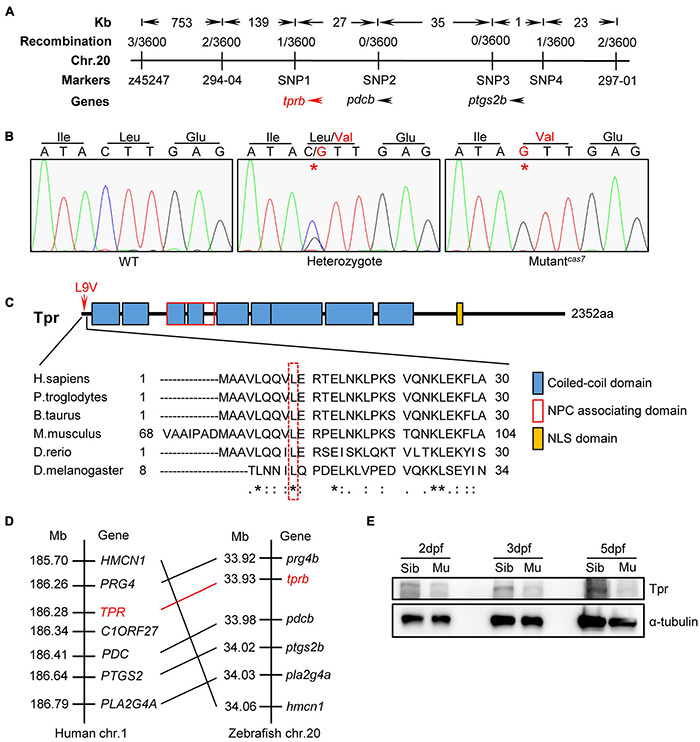
Positional cloning of mutant^*cas*7^. **(A)** Genetic mapping of the *cas7* region on chromosome 20. Bulk segregation analysis positioned *cas7* mutation to Chr. 20. Fine mapping using SSLPs narrowed down the region between markers SNP1 and SNP4, including *tprb* and two other genes as indicated. **(B)** There is a C-to-G missense mutation in *tprb* gene in mutant^*cas*7^, which leads to leucine to valine (L9V) at ninth amino acid in Tpr protein. **(C)** The ninth amino acid leucine of Tpr is highly conserved from human to fruit fly (* marked). **(D)** Comparison of the gene syntenic relationship between zebrafish *tprb* and human *TPR* loci. Seven genes, including *TPR*, are located within a genomic region on human chromosome 1. (Right) Seven zebrafish homologs of these genes are listed according to their map positions on chromosome 20 (Ensembl website). Mb, mega base. **(E)** Western blotting images of Tpr protein in whole embryo lysates of sibling and mutant^*cas*7^ embryos at the indicated time. All embryos were extracted for genomic DNA and genotyped by sequencing.

To elucidate the mechanism of erythroid maturation failure in mutant^*cas*7^, we carried out positional cloning. The mutant^*cas*7^ was first mapped on chromosome 20 by bulk segregation analysis (BSA). A high-resolution sequence length polymorphism (SSLP)–based mapping approach established the mutation within a 225-kb region between two markers 294-04 and 297-01 (Figure 2A). After sequencing all three candidate genes in this region, we found a C to G missense mutation in *tprb* gene in mutant^*cas*7^ (Figure 2B). Also, genomic sequencing of *tprb* gene also confirmed this result. This mutation yielded the leucine to valine at ninth amino acid (L9V) of Tpr protein and the leucine was highly conserved from human to fruit fly (Figure 2C). Among five commonly used laboratory zebrafish strains (Tu, WIK, AB, Shanghai, and Longfin), this point mutation was not found (Supplementary Figure 4), excluding the possibility of single-nucleotide polymorphism (SNP). The *tprb* locus on zebrafish chromosome 20 is syntenic to a region of human chromosome 1 that contains the *TPR* gene, based on the conserved location of neighboring orthologous gene pairs (*PRG4*, *PDC*, *PTGS2*, *PLA2G4A*, and *HMCN1*), suggesting that zebrafish *tprb* gene is an ortholog of human *TPR* (Figure 2D).

To explore the consequence of the point mutation to *tprb* gene, we customized an anti-Tpr antibody to examine the endogenous Tpr protein level. Western blot results showed that the expression of Tpr protein was markedly decreased over time in mutant^*cas*7^, and became undetectable at 5 dpf, suggesting that this point mutation may disrupt the function of *tprb* gene (Figure 2E).

### Missense Mutation in *tprb* Gene Is Responsible for Mutant^*cas7*^

**FIGURE 3:**
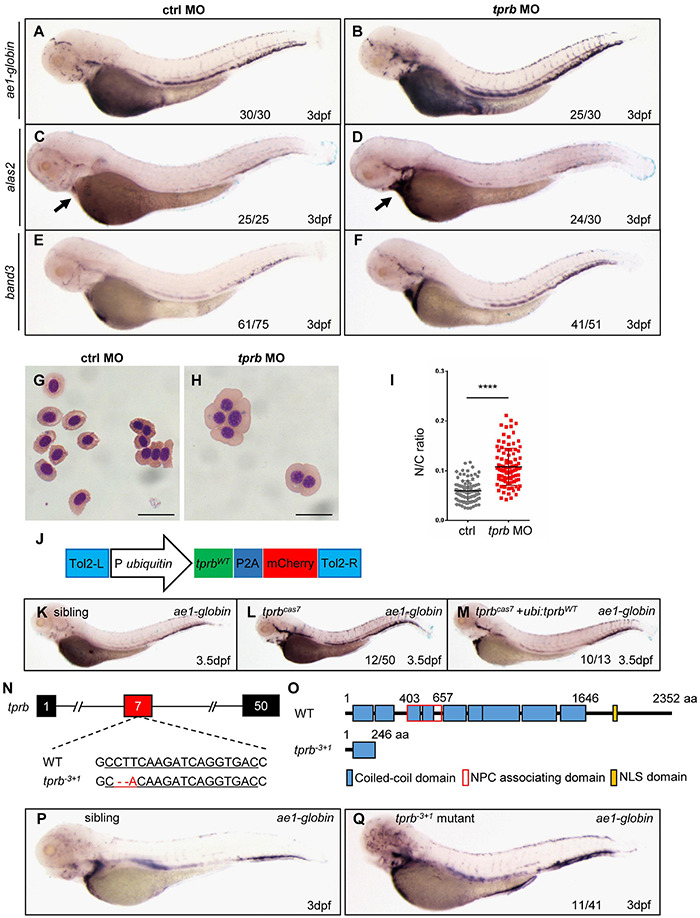
Mutation in *tprb* gene is responsible for mutant^*cas*7^ phenotypes. **(A–I)** Morpholino knocking down of *tprb* mutant^*cas*7^ phenocopies. WISH results of *ae1-globin* **(A,B)**, *alas2* **(C,D)**, and *band3* **(E,F)** expression in control and *tprb* morphants at 3 dpf. The percentages of indicated phenotype are listed at the bottom of each panel. Arrow in panel **(E,F)** indicates the *alas2* staining in heart region. **(G,H)** Giemsa staining for erythrocytes at 4 dpf in control and *tprb* morphants. More immature erythrocytes in *tprb* morphants are shown. Scale bars represent 10 μm. **(I)** Statistical analysis of the nucleus-to-cytoplasm (N/C) ratio in control and *tprb* morphants. *N* = 100, error bars represent SEM. ****p < 0.0001. **(J)** Construction of the plasmid used in Tol2-transposease-mediated rescue assay. **(K–M)** WISH analysis of *ae1-globin* expression in sibling, *tprb*^*cas7*^ mutants, and rescued embryos at 3.5 dpf. After WISH and photographing, all embryos were extracted for genomic DNA and genotyped by sequencing, then the rescue percentage was evaluated. The percentage of fully rescued mutant embryos is about 77% (10 out of 13 mutant embryos), while the rest are rescued partially. **(N,O)** Generation of *tprb^– 3^*^+^*^1^* mutant *via* CRISPR-Cas9 technique. The alignment of WT and mutated sequences is listed. The underlined sequence is gRNA target site. The sequencing result of *tprb* genomic DNA shows a 3-bp deletion and 1-bp addition at exon 7 **(N)**, which caused a premature stop codon leading to the production of a truncated 246-amino-acid Tpr protein **(O)**. **(P,Q)** WISH results of *ae1-globin* expression in sibling and *tprb^– 3^*^+^*^1^* mutant at 3 dpf. After WISH and photographing, all embryos were extracted for genomic DNA and genotyped by sequencing, then the mutant percentage was evaluated. The number of embryos in *tprb^– 3^*^+^*^1^* mutant incross clutch with the expression pattern as shown in the *tprb^– 3^*^+^*^1^* column and corresponding percentages are listed inside each panel.

To confirm the point mutation in *tprb* gene is responsible for mutant^*cas*7^ phenotypes, we injected a validated *tprb* ATG morpholino (MO) into one-cell-stage wild-type embryos to block the translation of endogenous *tprb* mRNA. Also, immunoblotting analysis confirmed the Tpr protein was poorly detectable in *tprb* morphants (Supplementary Figure 5A). WISH results showed that erythroid markers including *ae1-globin*, *alas2*, and *band3* were all upregulated (Figures 3A–F), while the other hematopoietic lineages were barely unchanged in *tprb* morphants (Supplementary Figures 5B–I). Consistent with WISH results, the real-time qPCR suggested the expression levels of *ae1-globin*, *alas2*, and *band3* were all enhanced, while other definitive hematopoietic cell markers (*cmyb, mpo, lyz, rag*, and *kdrl*) were unchanged (Supplementary Figure 5J).

To check whether and when the erythroid maturation was impaired, we have performed a different time course of Giemsa staining in circulating erythroid cells in control and *tprb* morphants. The result showed that before 2 dpf, the N/C ratio was not significantly changed between control and *tprb* morphants. Beginning at 3 dpf, the N/C ratio of *tprb* morphants was obviously increased, indicating that erythroid cell differentiation was inhibited from 3 dpf (Supplementary Figure 6B). The bigger nuclear area in erythroid cells and immature erythrocytes in *tprb* morphants could be observed at 3 and 4 dpf (Supplementary Figures 6A,C–J). Together with these data, Giemsa staining showed more immature erythrocytes in *tprb* morphants by morphological assessment and nucleo-cytoplasmic ratio computation (Figures 3G–I), indicating impaired erythroblast maturation in *tprb* morphants.

To further demonstrate our findings, rescue assay was performed by ectopic expression of wild-type *tprb* under the *ubiquitin* promoter in mutant^*cas*7^ (Figure 3J). The result showed that when Tpr protein was rescued in *tprb* mutants, the upregulated erythroid markers could be also largely rescued by wild-type *tprb* overexpression (Figures 3K–M and Supplementary Figure 7).

Moreover, we generated a second zebrafish *tprb^–3+1^* mutant by CRISPR-Cas9, which carried a frameshift mutation and premature stop codon in exon 7 and led to a truncated Tpr protein (Figures 3N,O). Consistent with phenotype of mutant^*cas*7^, the embryonic development of *tprb^–3+1^* mutant was normal before 3 dpf but died at 5–6 dpf, and WISH analysis of *ae1-globin* level was also significantly increased (Figures 3P,Q). Thus, results from MO phenocopy assay, wild-type *tprb* rescue assay, and same phenotype of *tprb^–3+1^* mutant strongly suggested that the C-to-G mutation led to *tprb* gene loss of function, which was causative for phenotype of mutant^*cas*7^. Hence, we rename the mutant as *tprb*^*cas7*^.

Given that Tpr is a structural protein that exists in every cell theoretically, we examined the temporal and spatial expression of *tprb* by WISH analysis. The result showed that *tprb* transcript was expressed as maternal mRNA and ubiquitously expressed during zebrafish embryogenesis (Supplementary Figure 8). To answer whether the localization of Tpr is in erythroid cells, we performed the Tpr immunofluorescence in *tprb* mutants and morphant erythroid cells at 3 dpf (Supplementary Figure 9). In erythroid cells from sibling and control morphant, Tpr protein is indeed located on the nuclear membrane (Supplementary Figures 9A,B,E,F). While in *tprb* mutants and morphants there was no fluorescence signal of Tpr protein in erythroid cells (Supplementary Figures 9C,D,G,H), indicating the endogenous Tpr protein was missing in *tprb* mutant or morphant, consisting with the WB result in Figure 2E.

### The Phenotype of *tprb*^*cas7*^ Mutant Is Independent on HIF Signaling Pathway

**FIGURE 4:**
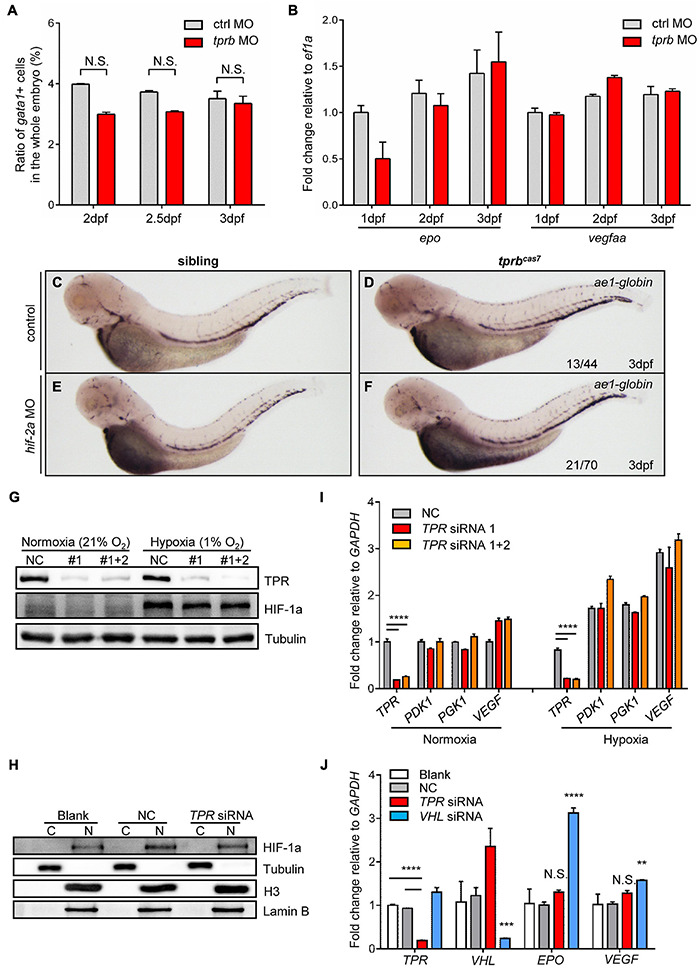
HIF signaling is not activated in *tprb*^*cas7*^ mutant. **(A)** The statistic results of the ratio of *gata1*^+^ cells sorted by flow cytometer in the whole embryo of control and *tprb* morphants by *Tg(gata1: DsRed; kdrl: EGFP)* transgenic line at different time points. **(B)** Quantitative PCR results of *epo* and *vegfaa* in control and *tprb* morphants at different time points. **(C–F)** WISH analysis of *ae1-globin* expression in sibling and *tprb*^*cas7*^ mutants at 3 dpf under the control and *hif-2a* morpholino **(E,F)** injection. **(G)** Immunoblotting analysis of TPR and HIF-1a protein level in whole HEK293T cells under normoxia and hypoxia conditions during *TPR* knockdown through siRNA. **(H)** Immunoblotting analysis of HIF-1a protein level in the nuclei of HEK293T cells under hypoxia condition after nuclear and cytoplasmic separation. C, cytoplasma; N, nucleus. **(I)** Quantitative PCR results of HIF target genes including *TPR, PDK1, PGK1*, and *VEGF* in HEK293T cells during *TPR* knockdown through siRNA. **(J)** Quantitative PCR results of HIF target genes including *EPO* and *VEGF* in Hep3B cells during *TPR* or *VHL* knockdown through siRNA under hypoxia condition. NC: negative control. #1: TPR siRNA 1; #1 + 2: TPR siRNA 1 + 2. Error bars represent SEM. N.S.: not significant; **p ≤ 0.01; ***p ≤ 0.001; ****p ≤ 0.0001.

It has been reported that HIF signaling pathway plays an important role in erythropoiesis, especially in erythrocytosis (Lee and Percy, 2011). Von Hippel-Lindau tumor suppressor (VHL) was the negative regulator of HIFs. The zebrafish *vhl* mutant displayed a hypoxia response, severe polycythemia, and immature erythropoiesis (van Rooijen et al., 2009). Also, results revealed global upregulation of both red and white hematopoietic lineages. Interestingly, we found that the phenotype of upregulation of erythroid genes and immature erythrocytes in *tprb*^*cas7*^ mutant was similar to reported *vhl* zebrafish mutant. Therefore, to reveal the molecular mechanism of erythroid maturation defect in *tprb*^*cas7*^ mutant, we explored whether the HIF signaling pathway was activated. First, flow cytometry analysis showed that the number of erythrocytes was not significantly changed in *tprb* morphants compared with control (Figure 4A and Supplementary Figure 10). Also, mRNA levels of *epo*, a HIF target gene involved in erythropoiesis (Bunn, 2013), and *vegfaa* were not elevated in *tprb* morphants (Figure 4B).

Furthermore, to compare the difference of phenotype in *tprb*^*cas7*^ mutant with embryos activated by HIF signaling, we established a *vhl* mutant by CRISPR-Cas9 as our positive control (Supplementary Figures 11A,B). To our surprise, WISH result of *ae1-globin* level was much more increased in *vhl* mutant than *tprb*^*cas7*^ mutant (Supplementary Figures 11C,D). Given that HIF-2a is mainly involved in the regulation of polycythemia (Lee and Percy, 2011; Metelo et al., 2015), we considered rescue experiment to alleviate the degree of polycythemia by using zebrafish *hif-2a* MO. WISH results showed that *hif-2a* MO could partially rescue the increased expression of *ae1-globin* in *vhl* mutant, which indicated the effectiveness of the *hif-2a* MO (Supplementary Figures 11E,F), but not in *tprb*^*cas7*^ mutant (Figures 4C–F). These results suggested that loss of *tprb* gene most likely did not cause the activation of HIF signal pathway.

Previous studies have demonstrated that HIF-1a protein is stabilized and enters the nucleus under hypoxic conditions, which is essential for hypoxic response (Koh and Powis, 2012). To further directly confirm whether loss of Tpr protein could result in the activation of HIF signaling, we analyzed the accumulation of HIF-1a protein and the expression of HIF target genes in human cultured cells under hypoxia condition. Western blot analysis in HEK293T cells showed that neither the amount of HIF-1a protein from whole cells nor nucleus was barely changed as *TPR* knock-down (Figures 4G,H). At the same time, the expression of several important HIF target genes including *PDK1*, *PGK1*, and *VEGF* also showed no significant change with or without TPR protein (Figure 4I). Similarly, we tested in human hepatocyte Hep3B cells and designed *VHL* siRNA as the positive control. Under hypoxic conditions, knocking down *VHL* could upregulate the expression of *EPO* and *VEGF* dramatically, whereas knocking down *TPR* had no significant change (Figure 4J). Collectively, the defective erythroid phenotype of *tprb*^*cas7*^ mutant was indeed independent on the activation of HIF signaling.

### Loss of Tpr Protein Enhances the Transcriptional Activity of Erythroid Genes

**FIGURE 5:**
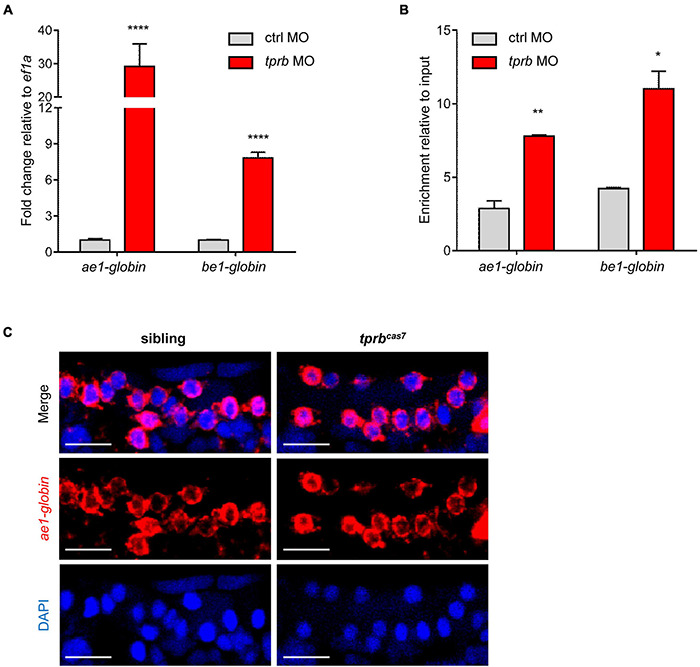
*tprb* inactivation enhances the transcriptional activity of erythroid genes. **(A)** Quantitative PCR results of *ae1-globin*, *be1-globin*, and *alas2* in erythrocytes after sorting an equal number of *gata1*^+^ cells in control and *tprb* morphants at 3 dpf. **(B)** Quantitative qChIP results of the binding enrichment of RNA polymerase II (Pol II) in *ae1-globin*, *be1-globin*, and *alas2* after sorting equal number of *gata1*^+^ cells. **(C)** Fluorescence *in situ* hybridization (FISH) results of *ae1-globin* mRNA localization in sibling and *tprb*^*cas7*^ mutants at 3 dpf. The nuclei were stained with DAPI (blue). Error bars represent SEM. N.S., not significant; *p < 0.05; **p < 0.01; ****p < 0.0001. Scale bars represent 20 μm.

As described previously, the number of erythrocytes in *tprb*^*cas7*^ mutant did not alter, leading us to investigate whether the increased staining of erythroid markers by WISH analysis in *tprb*^*cas7*^ mutant was caused by the upregulation of genes in every erythrocyte. To test this hypothesis, we injected the control and *tprb* MO into one-cell-stage embryos of Tg(gata1: DsRed) transgenic line separately. Then we sorted the same number of erythrocytes through *Tg(gata1: DsRed)* transgenic line and carried out real-time qPCR to analyze the expression levels of erythroid genes. As expected, the *ae1-globin* and *be1-globin* expression levels were dramatically increased in *tprb* morphants, especially for *ae1-globin* (Figure 5A).

To determine how Tpr deficiency triggered erythroid genes expression abnormally, we checked the transcriptional activity of these genes by quantitative chromatin immunoprecipitation (qChIP) experiment through RNA polymerase II (Pol II) antibody. ChIP results showed that the relative abundance of Pol II in the promoter region of *ae1-globin* and *be1-globin* in *tprb* morphants was dramatically higher than that of in control morphants (Figure 5B).

In yeast, Mlp1 and Mlp2 proteins (ortholog of mammalian Tpr protein) contribute to RNA surveillance by retention of unspliced mRNA in nucleus (Green et al., 2003; Galy et al., 2004) and downregulate gene expression in response to mRNA export defect (Vinciguerra et al., 2005). To analyze whether the mRNA export is blocked in erythrocytes in *tprb*^*cas7*^ mutant, we tested the cellular localization of *ae1-globin* mRNA. Fluorescent *in situ* hybridization result showed that the *ae1-globin* mRNA was exported out of the nucleus normally and distributed throughout the cytoplasm in both sibling and *tprb*^*cas7*^ mutant, indicating the mRNA export was normal (Figure 5C). These results taken together suggested that loss of Tpr can upregulate erythroid genes expression by enhancing their transcriptional activity, rather than the number of erythrocytes.

### Chromatin Condensation Defects Within Erythroid Nuclei in *tprb*^*cas7*^ Mutant

**FIGURE 6:**
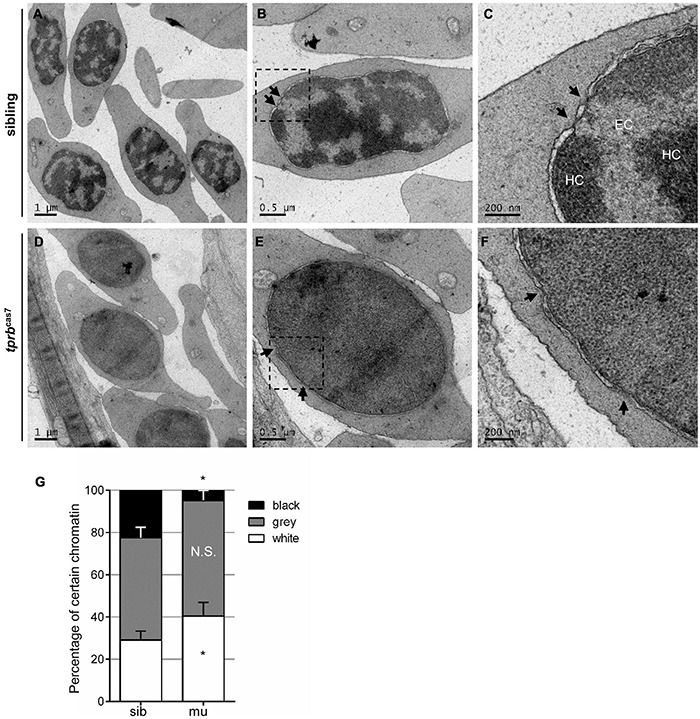
Chromatin condensation is defective in *tprb*^*cas7*^ mutant erythroid cells. **(A–F)** Representative transmission electron microscopy (TEM) images of erythrocytes from sibling **(A–C)** and *tprb*^*cas7*^ mutants **(D–F)** at 4 dpf. The images in panels **(C,F)** showed a higher magnification of the corresponding boxes **(B,E)**. Arrows indicate nuclear pores. EC, euchromatin; HC, heterochromatin. Scale bars are shown in each image. **(G)** Quantifications of certain chromatin with classified different color in sibling and *tprb*^*cas7*^ mutants at 4 dpf. Error bars represent SEM. N.S., not significant; *p < 0.05.

Chromatin condensation is essential for terminal erythropoiesis and erythroid maturation. To further explore how Tpr regulate the transcriptional activity of erythroid genes, we performed transmission electron microscopy to analyze the ultrastructural changes in the nucleus. It was found that at 4 dpf, there were distinct heterochromatin regions in most of the nuclei of erythrocytes in siblings, and the separation between euchromatin and heterochromatin was obvious (Figures 6A–C). Also, in *tprb*^*cas7*^ mutant, the boundary between them was hard to define precisely, presenting a “homogeneous” chromatin state (Figures 6D–F). Relying on the chromatin aggregation degree within the nuclei of erythrocytes, statistical analysis result showed that the proportion of euchromatin was significantly increased, whereas the heterochromatin percentage markedly decreased in *tprb*^*cas7*^ mutant (Figure 6G). Moreover, we also found the disruption of HEZs near NPCs in *tprb*^*cas7*^ mutant as previously reported (Krull et al., 2010). The aforementioned results indicated that the chromatin condensation in erythroid cells was impaired in *tprb*^*cas7*^ mutant.

Given Tpr is a structural protein, we analyzed chromatin structures in other tissue cells to test whether chromatin organization abnormality was a common phenomenon. The results showed that such heterochromatin blockage surprisingly only existed in erythrocytes and some hematopoietic cells, not in other tissue cells, such as neural cells, endothelial cells, and skeletal muscular cells (Supplementary Figure 12). Also, there was no significant difference in chromatin morphology in those tissue cells between sibling and *tprb*^*cas7*^ mutant (Supplementary Figure 12). Overall, these results suggested that Tpr deficiency might specifically disrupt the chromatin condensation and later on maturation in erythrocytes.

### TPR Is Also Important for Erythroid Differentiation in Humans

To test whether Tpr is also important for mammalian hematopoiesis, we knocked down *TPR* in human K562 cells (Supplementary Figure 13). The *TPR* shRNA efficiently knocked down TPR expression (Supplementary Figures 13A,B). The K562 line is composed of undifferentiated blast cells that are rich in glycophorin and may be induced to produce fetal and embryonic hemoglobin in the presence of hemin. The K562 cell membrane glycoproteins show many similarities with that of erythrocytes and, in particular, the cells synthesize glycophorin A (CD235a, also named GPA), which is found exclusively in human erythrocytes (Drexler et al., 2004). Therefore, the K562 cell appears to be an excellent tool for the study of human erythroid differentiation and globin gene expression.

We induced K562 erythroid differentiation by treated with hemin, to find TPR functions in erythroid differentiation. Expression of the glycophorin A (CD235a) on the surface of K562 cells was determined by direct immunofluorescence staining and flow cytometer. When treated with hemin for 48 h, the expression of CD235a on the surface showed a significant increase in scramble shRNA K562 cells; however, the expression of CD235a in *TPR* knock-down K562 cells was not obviously enhanced (Supplementary Figures 13C,D). We also checked globin expression when *TPR* is knocked down. The α globin and β globin upregulated in *TPR* knock-down cells, while γ globin and δ globin was not obviously changed (Supplementary Figure 13E). When treated with hemin, the expressions of α globin, β globin, and γ globin were all increased (Supplementary Figure 13E). These data indicated that *TPR* knock-down caused blocking of erythroid differentiation, although the globin expression increased largely in K562 cell line.

## Discussion

Here, we reported a novel zebrafish *tprb*^*cas7*^ mutant with defect in erythroid maturation. Genetic analysis revealed that the missense mutation in *tprb* gene leads to Tpr protein loss of function, which was causative for *tprb*^*cas7*^ mutant phenotypes. Further studies found that Tpr was essential for the organization and maintenance of chromatin condensation and erythroid gene regulation during terminal erythropoiesis, and the function of TPR in erythropoiesis is highly conserved in mammalian.

In this study, we confirmed that the point mutation in *tprb* gene would lead to Tpr protein loss of function by *tprb* knockdown, rescue, and knockout experiments. Zebrafish Tpr protein contains 2,352 amino acids. It is interesting to find how the single base mutation results in whole protein inactivation. By detecting the amount of Tpr protein at different stages, we found that the level of Tpr was gradually decreased in *tprb*^*cas7*^ mutant and almost disappeared from 3 dpf (Figure 2E). This result indicates that the missense mutation might affect the stability of Tpr proteins and accelerate their degradation, leading to the loss of function finally. In addition, the erythroid phenotype of *tprb*^*cas7*^ heterozygotes was checked, and no defect was observed. We propose that the mutation type of Tpr (L9V) has no dominant negative effect.

The increased staining of WISH generally shows the increased expression level of the genes. We found that *tprb* knockdown did not change the number of erythrocytes compared with control, although the expressions of erythroid markers (*ae1-globin*, *alas2*, and *band3*) were all increased by WISH staining. Thus, our results showed that loss of Tpr could upregulate the expression of erythroid genes by enhancing their transcriptional activity.

Also, we confirmed that the phenotype of *tprb*^*cas7*^ mutant was independent on HIF signaling pathway mainly through four reasons: (1) the number of erythrocytes was not increased; (2) the expression of HIF target genes was not upregulated; (3) *hif-2a* MO could partially rescue the WISH result of *ae1-globin* in *vhl* mutant, rather than in *tprb*^*cas7*^ mutant; (4) in human cells, there was no significant change in the accumulation of HIF-1a protein and expression of HIF target genes as *TPR* knockdown.

Although the expression of NPC as the structural protein has no tissue specificity, its tissue-specific function has been widely reported (Raices and D’Angelo, 2012). In this study, we found that the chromatin condensation and organization were specifically disrupted in erythrocytes in *tprb*^*cas7*^ mutant. On the one hand, this may be the process of nuclear condensation and globally chromatin aggregation specific to erythrocytes, and erythroid terminal differentiation highly depends on this. Therefore, the erythroid cells in *tprb*^*cas7*^ mutant are more sensitive to chromatin changes. On the other hand, studies have identified different molecules involved in chromatin reorganization during terminal differentiation in different types of cells. Such as in terminally differentiating myotubes, methyl CpG–binding protein MeCP2 and MBD2 are important for the aggregation of pericentric heterochromatin (Brero et al., 2005). Our finding that the chromatin condensation in muscular cells was not affected in *tprb*^*cas7*^ mutant is consistent with the previous research.

In different species, there are various molecular mechanisms involved in chromatin condensation during terminal erythropoiesis. In chicken erythrocytes, two architectural factors, linker histone H5 and nuclear serpin MENT, accumulate at repressed chromatin domains and promote chromatin condensation (Verreault and Thomas, 1993; Istomina et al., 2003). Also, in mammalian erythrocytes, histone deacetylation and caspase-3 may be the greatest players (Zermati et al., 2001; Popova et al., 2009; Ji et al., 2011; Zhao et al., 2016). In this study, we first showed that nucleoporin Tpr, which faces the nucleoplasm, was essential for chromatin condensation and erythroid maturation in zebrafish embryogenesis. Also, we found that the human TPR was important for K562 erythroid cell maturation. It is still intriguing to explore whether the histones and their modifications are involved in this process and how they can corporate with Tpr. One clue is that Tpr can specifically interact with histone H1.1 and H1.2 to regulate the stability of these replication-dependent linker histones (Zhang et al., 2016).

At present, there are some human diseases associated with TPR. In several different cancers, TPR is present through fusion genes, which is formed by coiled-coil motif in TPR with some kinase partner genes such as *MET* (gastric cancer) (Soman et al., 1991), *NTRK1* (thyroid carcinoma) (Greco et al., 1992), *FGFR1* (myeloproliferative syndromes) (Li et al., 2012), and *ALK* (lung adenocarcinoma) (Choi et al., 2014). In all fusion proteins, TPR N-terminal coiled-coil domain is maintained and fused to the partner kinase domain, resulting to allow dimerization that aberrantly activates kinases and drives the cancer progression. In addition, patients with Hutchinson–Gilford Progeria Syndrome (HGPS) premature aging had abnormal cytoplasm localization of TPR protein. HGPS is caused by a mutation in *LMNA* gene that generates a mutant lamin A protein, which is a major component of the nuclear lamina. Fibroblasts from HGPS patients have defects in the Ran GTPase system that cause a nuclear import defect in TPR and mislocalized in the cytoplasm, not the nuclear side of the nuclear pore complex (Snow et al., 2013). However, it is not clear how the abnormal localization of TPR affects the development of the HGPS.

Previous studies have reported that the chromatin structure can determine the expression of specific genes (Deng et al., 2012; Deng et al., 2014). During terminal erythropoiesis, nuclear and chromatin are gradually condensed and associated with wide downregulation of gene expression (Wong et al., 2011). In *tprb*^*cas7*^ mutant, chromatin condensation was destroyed, accompanied by a significant increase in euchromatin ratio. This was consistent with the increased total mRNA amount as *tprb* knockdown (data not shown). However, the relationship between chromatin organization and gene expression is still unclear in *tprb*^*cas7*^ mutant. It is necessary to investigate how the chromatin state and the transcriptome changed.

NPCs have emerged as a crucial regulator of chromatin organization and gene expression (Ibarra and Hetzer, 2015). As an architectural component of NPCs, although Tpr can directly bind to 25% of the genome, most functions of Tpr depend on its interaction with Nup153 to locate at the NPC (Vaquerizas et al., 2010). It is believed that the nuclear envelope is associated with heterochromatin, whereas NPCs are surrounded by euchromatin. The maintenance of these heterochromatin exclusion zones was shown to involve Tpr (Krull et al., 2010). And recently, Tpr is reported to be necessary for SAHF formation, which also relies on its association with the NPC (Boumendil et al., 2019). Nup62 is an element of central channel of NPC (Grossman et al., 2012). We found that the WISH result of *ae1-globin* was increased in zebrafish *nup62l* mutant (Supplementary Figure 14), which was identical to *tprb*^*cas7*^ mutant. It was suggested that the function of Tpr in regulation of erythroid gene expression might be associated with NPC. It will be interesting to clarify the precise mechanism of Tpr in maintenance of chromatin condensation and gene expression. Also, our study would not only uncover the important role of Tpr in chromatin condensation but also facilitate *ex vivo* production of erythrocytes and regulation of chromatin status to change the cell fate.

## Materials and Methods

### Zebrafish Feeding and Maintenance

The zebrafish facility and study were approved by the Animal Research Advisory Committee of Institute of Nutrition and Health, SIBS, CAS, and zebrafish were maintained according to the guidelines of the Institutional Animal Care and Use Committee. Wild-type (WT) zebrafish strains Tubingen (Vinciguerra et al., 2005) and WIK, the transgenic zebrafish line *Tg(gata1: DsRed)* (Traver et al., 2003), and *Tg(kdrl: EGFP)* (Cross et al., 2003) were described previously. To prevent melanin pigment formation, embryos were incubated in egg water containing 0.045% 1-phenyl-2-thiourea (PTU; Sigma) after 1 dpf and the egg water was changed every day. The embryos were collected at the desired stages (Kimmel et al., 1995).

### ENU Mutagenesis and Positional Cloning

ENU mutagenesis and positional cloning were performed as previously described (Bahary et al., 2004). The *cas7* (Tu background) allele was mapped by out-crossing Tu background heterozygous fish into polymorphic WIK background wild-type strain. The mutant^*cas*7^ was first mapped on chromosome 20 by linked SSLP markers by bulk segregation analysis (BSA) (Knapik et al., 1998; Shimoda et al., 1999). Simple sequence-length polymorphism (SSLP) markers used for BSA were selected from the Massachusetts General Hospital Zebrafish Server website (MGH)^1^. Fine mapping using mainly SSLP markers was carried out to narrow down the genetic interval within a 225-kb region between two markers 294-04 and 297-01. The cDNAs of three candidate genes in the interval were cloned and sequenced from mutants. The sequence *of tprb* gene in mutants^*cas*7^ had a C to G missense mutation, while other genes were normal. Also, the C to G missense mutation was confirmed by sequencing genomic DNA. All primers used for this study are provided in Supplementary Table.

### Microinjection and CRISPR-Cas9 Mutagenesis

Morpholino oligonucleotides (MOs) used for microinjection were ordered from Gene Tools. The MOs were microinjected into one-cell-stage embryos as previously described (Nasevicius and Ekker, 2000). Transient *tprb* transgene construct within Tol2 vectors (40 pg) was microinjected into one-cell-stage embryos with Tol2 transposase mRNA (50 pg). CRISPR-Cas9–mediated generation of zebrafish mutants (*tprb^–3+1^* and *vhl*) were performed as previously described (Xiao et al., 2013). The gRNAs (50 pg) and Cas9 protein (500 pg) were co-microinjected into one-cell-stage embryos.

### Plasmid Construction

The zebrafish cDNA of *tprb* gene was amplified from reverse transcription products and cloned into pCS2*^+^* vector with *Bam*HI and *Eco*RI restriction sites. To construct Tol2 transgenesis vectors, the *ubiquitin* promoter followed by P2A and in-frame mCherry was cloned into modified Tol2 backbone. Also, the zebrafish *tprb*^*WT*^ was amplified from *tprb*-pCS2^+^ plasmid and inserted between the *ubiquitin* promoter and P2A with *Bam*HI restriction site. We changed several bases of the first 30 bases in the *tprb*^*WT*^ gene, to mismatch the *tprb* ATG MO sequence. Changes in these bases would not affect the *tprb* amino acid sequence, for these bases are all changes in synonymous amino acids.

### Conventional Whole-Mount *in situ* Hybridization and FISH Analysis

The antisense RNA probes were transcribed *in vitro* by T3 or T7 polymerase (Ambion) with Digoxigenin RNA Labelling Mix (Roche). Conventional and fluorescence whole-mount *in situ* hybridization (WISH and FISH) was described previously (Jowett and Lettice, 1994). In WISH, NBT/BCIP (Sigma) was used for staining and images were captured by Olympus SZX16 microscope. In FISH, embryos were stained with Cy5 (TSA system; Perkin Elmer) and DAPI (1:500; Beyotime) and imaged by Olympus FV1000 confocal microscope.

### Giemsa Staining

Zebrafish embryos at indicated time points were anesthetized in calcium-free and magnesium-free PBS (pH 7.4) containing 0.02% tricaine (Sigma) and 1% BSA. Circulating blood cells were collected by cutting tails and cytospined onto slides by centrifugation at 1,000 rpm for 3 min. Then the cells were air-dried and stained by Giemsa solutions (Nanjing Jiancheng) according to the manufacturer’s protocol. Images were finally captured by Zeiss Axio Imager A2 microscope. Nuclear area, and nuclear and cytoplasmic diameters of at least 100 randomly selected erythrocytes were measured by using ImageJ software, and nucleo-cytoplasmic ratio was calculated through spherical volume formula.

### Flow Cytometry, RNA Extraction, and Quantitative PCR Analysis

The analysis and sorting of *gata1*^+^ erythrocytes from *Tg(gata1: DsRed; kdrl: EGFP)* embryos were carried out with flow cytometer (Beckman) as previously described (Traver et al., 2003). For total RNA extraction, 10–20 zebrafish embryos or the sorted erythrocytes or cultured human cells were dissolved by TRIzol (Invitrogen), and then transcribed into cDNA by PrimerScript RT Master Mix (TaKaRa). The SYBR Green Real-time PCR Master Mix (TOYOBO) was used to perform quantitative PCR system with ABI Q7 real-time PCR instrument. The primers are listed in Supplementary Table.

### Cell Culture and siRNA Transfection

HEK293T and Hep3B cells were cultured in DMEM with 10% fetal bovine serum (FBS) and 1% penicillin and streptomycin. O_2_ (1%) was generated by flushing a 94% N_2_/5% CO_2_ mixture into the incubator and sustained for 48 h until cell confluence. For siRNA transfection, Lipofectamine RNAiMAX Reagent (Invitrogen) was used following the manufacturer’s protocol.

### Protein Extraction and Immunoblotting

To extract the protein from zebrafish embryos, the embryos were de-membraned and de-yolked first, then homogenized in lysis buffer [20 mM Tris–HCl (pH 7.4), 150 mM NaCl, 5 mM EDTA, 10% glycerol, 0.1% Triton X-100, and protease inhibitor cocktail] and boiled with 2 × SDS buffer for 10 min. To obtain the protein from cultured cells, the cells were homogenized directly with 2 × SDS buffer and boiled for 10 min. Nuclear and cytoplasmic extracts were prepared from cultured cells with Nuclear and Cytoplasmic Extraction Kit (CWBIO) according to the manufacturer’s instruction. The immunoblotting was carried out as previously described (Gao et al., 2015), with rabbit anti-Tpr antibody (immunized by 461-710 amino acids of zebrafish Tpr protein; Abclonal), mouse anti-a-tubulin (Sigma), mouse anti-HIF-1a (NOVUS), rabbit anti-TPR (human) (Bethyl Laboratory), goat anti-Lamin B (Santa Cruz), mouse anti-H3 (CST), and goat-anti-rabbit/mouse and donkey-anti-goat secondary antibodies (LIANKE).

### Immunofluorescence

Zebrafish embryos at indicated time points were anesthetized in calcium-free and magnesium-free PBS (pH 7.4) containing 0.02% tricaine (Sigma) and 1% BSA. Circulating blood cells were collected by cutting tails and cytospined onto slides by centrifugation at 1,000 rpm for 3 min. Immunofluorescence was carried out as previously described (Gao et al., 2015). The cells were stained with rabbit anti-Tpr antibody, fluorescence was detected with goat-anti-rabbit Alexa Fluor 488 secondary antibody (Invitrogen) and DAPI (Beyotime) was used for nucleus staining.

### Low Cell Quantitative ChIP

Quantitative ChIP was performed as previously described (Wardle et al., 2006; Wong et al., 2011) with modifications. Briefly, about 300,000 *gata1*^+^ erythrocytes from Tg(*gata1*: DsRed) embryos at 3 dpf were sorted and cross-linked with 1% formaldehyde for 10 min at room temperature, followed by glycine (0.125 M) treatment for 5 min. Then the cells were homogenized in lysis buffer [50 mM Tris–HCl (pH8.0), 10 mM EDTA (pH8.0), 0.2% SDS, and Cocktail proteinase inhibitor] and sonicated by Bioruptor to yield fragments. Each sample was incubated with RNA Pol II antibody (clone CTD4H8, Millipore) overnight at 4°C, followed by washing and elution with magnetic beads, and reverse cross-linked for 6 h at 65°C. DNA was purified using MinElute PCR Purification Kit (QIAGEN). The primers used in qChIP are shown in Supplementary Table.

### Transmission Electron Microscopy

For sample preparation, the tails of zebrafish embryos at 4 dpf were fixed with PBS solution containing 2% paraformaldehyde and 2.5% glutaraldehyde overnight at 4°C, followed by 2% osmium tetroxide. The tails were dehydrated in ethanol and infiltrated in acetone, then embedded in epon 812, and sections were analyzed using a FEI Tecnai G2 Spirit electron microscope. Quantification of certain chromatin was performed as previously described (Baarlink et al., 2017) with modifications. Briefly, each nucleus of erythrocyte was manually segmented and generated a binary mask by ImageJ. Then, the certain chromatin state was classified by the different gray threshold values. Finally, the proportion of certain chromatin was subsequently analyzed by measuring the chromatin area and nucleus area.

### Construction TPR Knockdown K562 Cell Line

The shRNA sequences for human *TPR* gene knockdown and the scrambled control shRNA sequence are supplied in Supplementary Table. The shRNA sequences were cloned into pLSLG lentiviral vector, which contained the EGFP for detecting transfection efficiency. The respective lentiviral vectors and helper vectors were transfected to 293T cells for viral packaging. Then 60 h after transfection, virus was collected to infect K562 cells in the presence of 10 mg/ml polybrene (Sigma-Aldrich). After 4 days, the cells were used in the experiments as described.

### Hemin Induced Erythroid Differentiation in Human K562 Cell Line

The K562 cells were treated with hemin (Sigma) at 0.04 mM for 48 h to induce erythroid differentiation. For immune-fluorescence staining, 1 × 10^6^ cells suspended into PBS were simultaneously incubated for 30 min of anti-APC-conjugated CD235a at 4°C avoiding light. The cells were then washed in PBS to remove unbound antibody for immediate flow cytometry analysis.

### Statistical Analysis

Statistical analysis was performed by GraphPad Prism 6 software using two-tailed Student’s *t*-test and *p* value of less than 0.05 was considered significant. Error values were calculated by SEM.

## Data Availability Statement

The raw data supporting the conclusion of this article will be made available by the authors, without undue reservation.

## Ethics Statement

The animal study was reviewed and approved by the Animal Research Advisory Committee of Institute of Nutrition and Health, SIBS, CAS.

## Author Contributions

SW, KC, WP, and XJ designed the research, analyzed the data, and wrote the manuscript. SW, KC, and TX performed the experiments. KM, LG, CF, WZ, CJ, CR, MD, and YC assisted with the experiments. CJ, XJ, LG, KM, YC, and MD contributed to the forward genetics screen. WP and YZ gave suggestions for experiment design. All authors contributed to the article and approved the submitted version.

## Conflict of Interest

The authors declare that the research was conducted in the absence of any commercial or financial relationships that could be construed as a potential conflict of interest.

## Publisher’s Note

All claims expressed in this article are solely those of the authors and do not necessarily represent those of their affiliated organizations, or those of the publisher, the editors and the reviewers. Any product that may be evaluated in this article, or claim that may be made by its manufacturer, is not guaranteed or endorsed by the publisher.

## Footnotes

1
http://zebrafish.mgh.harvard.edu

## Supplementary Material

Supplementary Figure 1 Developmental serial analysis of α*e1-globin* expression in mutant^*cas*7^. Embryos were fixed at time points as indicated, followed by WISH analysis of α*e1-globin* expression. Expression of α*e1-globin* is shown in siblings and mutants at 36 hpf **(A,B)**, 2 dpf **(C,D)**, 3 dpf **(E,F)**, and 4 dpf **(G,H)**. The number and percentage of het-het incross embryos with the *in situ* pattern are listed at the bottom of the mutant^*cas*7^ panels.

Supplementary Figure 2 WISH analysis of hematopoietic lineages at 3/4 dpf. WISH analysis of *cmyb* **(A,B)**, *mpx* **(C,D)**, *lyz* **(E,F)**, and *rag-1* **(G,H′)** expression shows no significant difference between sibling and mutant^*cas*7^ at 3/4 dpf. After WISH and photographing, all embryos were extracted for genomic DNA and genotyped by sequencing, then the mutant percentage was evaluated. The percentages of embryos in a het-het incross clutch with the expression pattern as shown in the mutant^*cas*7^ column is listed at the bottom of each panel. **(I)** The relative expression of α*e3-globin*, β*e1-globin*, β*e2-globin*, *hebp2*, *sdhb*, *fth1a*, *cmyb*, *mpo*, *lyz*, *kdrl*, and *rag1* in sibling and mutant^*cas*7^ embryos at 3 dpf. Error bars represent SEM. ns, not significant; **p* ≤ 0.05; ^∗∗^*p* ≤ 0.01; ^∗∗∗^*p* ≤ 0.001.

Supplementary Figure 3 Normal primitive hematopoiesis and vascular morphogenesis in mutant*^*cas7*^.* Sibling and mutant^*cas*7^ embryos were fixed at 22 or 26 hpf, followed by WISH analysis of *scl*, *gata1*, *pu.1*, *mpx*, and *kdrl* expression. Expressions of *scl* **(A,B)**, *gata1* **(C,D)**, *pu.1* **(E,F)**, *mpx* **(G,H)**, and *kdrl* **(I,J)** show no significant difference between wild-type siblings and mutants. Insets in panels **(E,F)** show dorsal views of the head staining region. After WISH and photographing, all embryos were extracted for genomic DNA and genotyped by sequencing, then the mutant percentage was evaluated. The number and percentage of het-het incross embryos with the *in situ* pattern are listed at the bottom of the mutant^*cas*7^ panels.

Supplementary Figure 4 The point mutation (C-G) in mutant^*cas*7^ was not found in all five wild-type strains, indicating that C-G transition at the site is not a SNP. Sequencing electropherogram of the mutation site of *tprb* genomic DNA from five wild-type strains. Genomic DNA were extracted each from 20 to 30 embryos of TU, AB, WIK, Longfin, and Shanghai strains, and then sequenced for *tprb* gene. This point mutation (C-G) in mutant^*cas*7^ was not found in all five wild-type strains.

Supplementary Figure 5 WISH analysis of hematopoietic lineages in *tprb* morphants at 3/4 dpf. **(A)** Representative immunoblotting images of Tpr in control and *tprb* morphants. WISH analysis of *cmyb* **(B,C)**, *mpx* **(D,E)**, *lyz* **(F,G)**, and *rag-1* **(H,I′)** expression show no significant difference between control and *tprb* morphants at 3/4 dpf. After WISH and photographing, all embryos were extracted for genomic DNA and genotyped by sequencing, then the mutant percentage was evaluated. The percentage of indicated phenotype is listed at the bottom of each panel. **(J)** The relative expression of α*e1-globin*, *alas2*, *band3*, α*e3-globin*, β*e1-globin*, β*e2-globin*, *gata1*, *hebp2*, *sdhb*, *fth1a*, *cmyb*, *mpo*, *lyz*, *kdrl*, and *rag1* at 3 dpf in *tprb* morphants. Error bars represent SEM. ns, not significant; ^∗∗^*p* ≤ 0.01; ^∗∗∗^*p* ≤ 0.001.

Supplementary Figure 6 Giemsa staining in circulating erythroid cells in control and *tprb* morphants at different time stages. **(A)** Quantitative analysis of each erythroid cell nuclear area. **(B)** Quantitative analysis of nucleus-to-cytoplasm (N/C) ratio. Error bars represent SEM. ns, not significant; *****p* ≤ 0.0001; ^∗∗^*p* ≤ 0.01; ^∗∗∗^*p* ≤ 0.001. **(C–G)** Representative images of Giemsa staining in circulating erythroid cells at different stages: 30 hpf **(C,D)**, 2 dpf **(E,F)**, 3 dpf **(G,H)**, and 4 dpf **(I,J)**. Scale bars represent 20μm.

Supplementary Figure 7 The result of *tprb*^*cas7*^ rescue experiment at 4 dpf. Transient expression of wild-type *tprb* under the *ubiquitin* promoter in mutant^*cas*7^ could rescue the phenotypes. Mutant^*cas*7^ embryos at one-cell stage were injected the tol2 plasmid with *tprb*^*WT*^ expression driven by ubiquitin promoter. Then embryos with mCherry fluorescence would be selected for downstream analysis. At 4 dpf, every embryo was cut off in head for genotyping, then the trunk and tail of the embryo was taken for RNA or protein extraction. After genotyping the mutant embryos, we pooled three to five of the same phenotype embryo RNA extraction and took the qPCR experiments. **(A)** The relative expression of α*e1-globin*, *alas2*, *band3*, α*e3-globin*, β*e1-globin*, and β*e2-globin* at 4 dpf. Error bars represent SEM. ^∗∗^*p* ≤ 0.01; ^∗∗∗^*p* ≤ 0.001. **(B)** Representative immunoblotting images of Tpr in *tprb* mutants rescued by tol2 plasmid with *tprb*^*WT*^ expression.

Supplementary Figure 8 Expression pattern of *tprb* mRNA during zebrafish embryogenesis. **(A–J)** WT (TU strain) embryos were fixed at the time points as indicated in each panel, followed by WISH analysis of *tprb* mRNA expression.

Supplementary Figure 9 The endogenous Tpr protein is located on the nuclear membrane and Tpr protein is missing in *tprb* mutant or morphant. Representative images of Tpr immunofluorescence in sibling **(A,B)** and mutants **(C,D)** at 3 dpf. All embryos were extracted for genomic DNA and genotyped by sequencing. Representative images of Tpr immunofluorescence in control morphants **(E,F)** and *tprb* morphants **(G,H)** at 3 dpf. Scale bars represent 20 μm.

Supplementary Figure 10 Flow cytometry analysis of erythrocytes from control and *tprb* morphants. Flow cytometry analysis of *gata1*^+^ cells from *Tg(gata1: DsRed; kdrl: EGFP)* transgenic line in control and *tprb* morphants at different time points. The ratio of *gata1*^+^ erythrocytes between control and *tprb* morphants was similar.

Supplementary Figure 11 Generation and phenotype characterization of zebrafish *vhl* mutant. **(A,B)** Generation of *vhl* mutant *via* CRISPR-Cas9 technique. The alignment of WT and mutated sequences is listed. The underlined sequence is *vhl* gRNA target site. The sequencing result of *vhl* genomic DNA showed changed bases (shown in red) at exon 1 (A), which caused a premature stop codon leading to the production of a truncated 31-amino-acid Vhl protein **(B)**. **(C–F)** WISH analysis of *ae1-globin* expression in sibling and *vhl* mutants at 3 dpf under the control and *hif-2a* morpholino **(E,F)** injection, indicating that the *hif-2a* MO can partially rescue the increased expression of *ae1-globin* in *vhl* mutant.

Supplementary Figure 12 The other tissue cells in *tprb*^*cas7*^ mutant have normal chromatin organization. **(A–F)** Representative TEM images of chromatin organization within the tissue cells show no obvious difference between sibling and *tprb*^*cas7*^ mutants at 4 dpf, including neural cells **(A,B)**, endothelial cells **(C,D)**, and muscular cells **(E,F)**.

Supplementary Figure 13 TPR is also important for erythroid differentiation in mammalian. **(A)** Representative immunoblotting images of TPR shRNA and scramble shRNA in K562 cell line. Tubulin serves as the loading control. **(B)** The relative expression of *TPR* in different groups. **(C)** Representative FACS images of the ratio of CD235a-positive cells with or without hemin treatment. **(D)** Quantitative analysis of panel **(C)**. **(E)** The relative expression of α/β/γ/δ-globins with or without hemin treatment. Error bars represent SEM. ns, not significant; **p* ≤ 0.05; ^∗∗^*p* ≤ 0.01; ^∗∗∗^*p* ≤ 0.001.

Supplementary Figure 14 Zebrafish *nup62l* mutants represent similar phenotype with *tprb*^*cas7*^ mutants. **(A,B)** WISH analysis of *ae1-globin* show the increased expression in *nup62l* mutants compared with sibling at 3.5 dpf. The number and percentage of het-het incross embryos with the *in situ* pattern in a clutch are listed at the bottom of the mutant panels.
