# Corrigendum: Tpr deficiency disrupts erythroid maturation with impaired chromatin condensation in zebrafish embryogenesis

**DOI:** 10.3389/fcell.2024.1409433

**Published:** 2024-04-24

**Authors:** Shuang Wu, Kai Chen, Tao Xu, Ke Ma, Lei Gao, Cong Fu, Wenjuan Zhang, Changbin Jing, Chunguang Ren, Min Deng, Yi Chen, Yi Zhou, Weijun Pan, Xiaoe Jia

**Affiliations:** ^1^ Shanghai Institute of Nutrition and Health, Chinese Academy of Science, Shanghai, China; ^2^ Central Laboratory, Qingdao Agricultural University, Qingdao, China; ^3^ State Key Laboratory of Medical Genomics, Ruijin Hospital, School of Medicine, Shanghai Jiao Tong University, Shanghai, China; ^4^ Stem Cell Program, Hematology/Oncology Program at Children’s Hospital Boston, Harvard Medical School, Boston, MA, United States; ^5^ Inner Mongolia Key Laboratory of Hypoxic Translational Medicine, Baotou Medical College, Baotou, China

**Keywords:** erythrocytes, erythroid maturation, chromatin condensation, zebrafish, Tpr

In the published article, there was an error in the **Funding statement**. An essential source of funding that significantly supported the work was inadvertently omitted from the original Funding statement. The original Funding statement was written as: “This work was supported by National Natural Science Foundation of China (81901918, 81660204 to XJ, 31571505 to WP) and Inner Mongolia Science Foundation (2019MS08060); and Innovative and Entrepreneurial Talents in the “Prairie Talents” Project of Inner Mongolia (Q2017047), local science and technology projects guided by the central government (2020ZY0040), and CAS “Light of West China” Program to XJ.” The correct Funding statement appears below.

