# Supplementary figures and images for: Tpr Deficiency Disrupts Erythroid Maturation With Impaired Chromatin Condensation in Zebrafish Embryogenesis
Source: Front Cell Dev Biol. 2021 Oct 13;9:709923. doi: 10.3389/fcell.2021.709923 (PMC8548687; doi:10.3389/fcell.2021.709923)

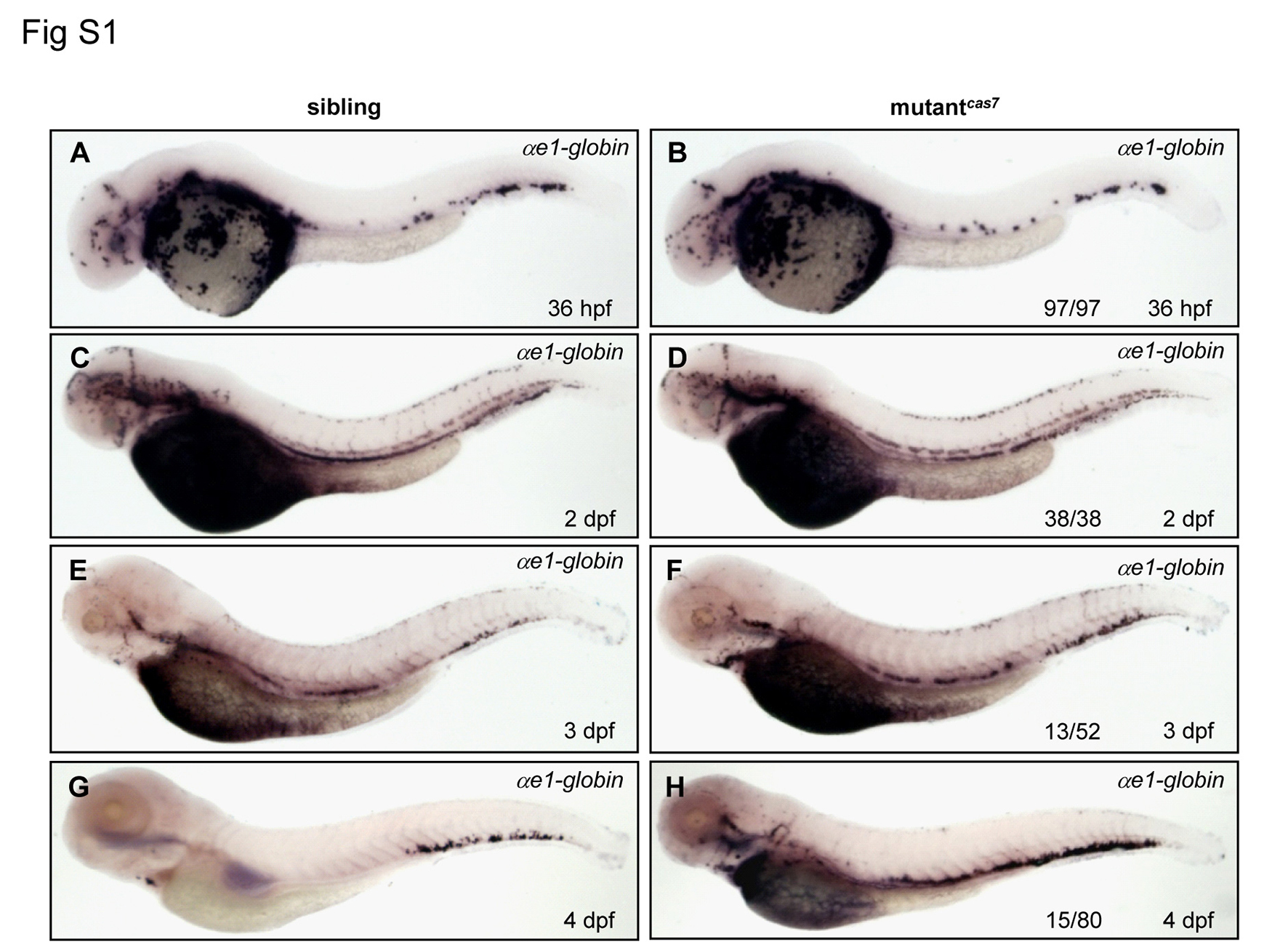

Supplement: Supplementary Figure 1 — Developmental serial analysis of αe1-globin expression in mutantcas7. Embryos were fixed at time points as indicated, followed by WISH analysis of αe1-globin expression. Expression of αe1-globin is shown in siblings and mutants at 36 hpf (A,B), 2 dpf (C,D), 3 dpf (E,F), and 4 dpf (G,H). The number and percentage of het-het incross embryos with the in situ pattern are listed at the bottom of the mutantcas7 panels. [file Image_1.JPEG]

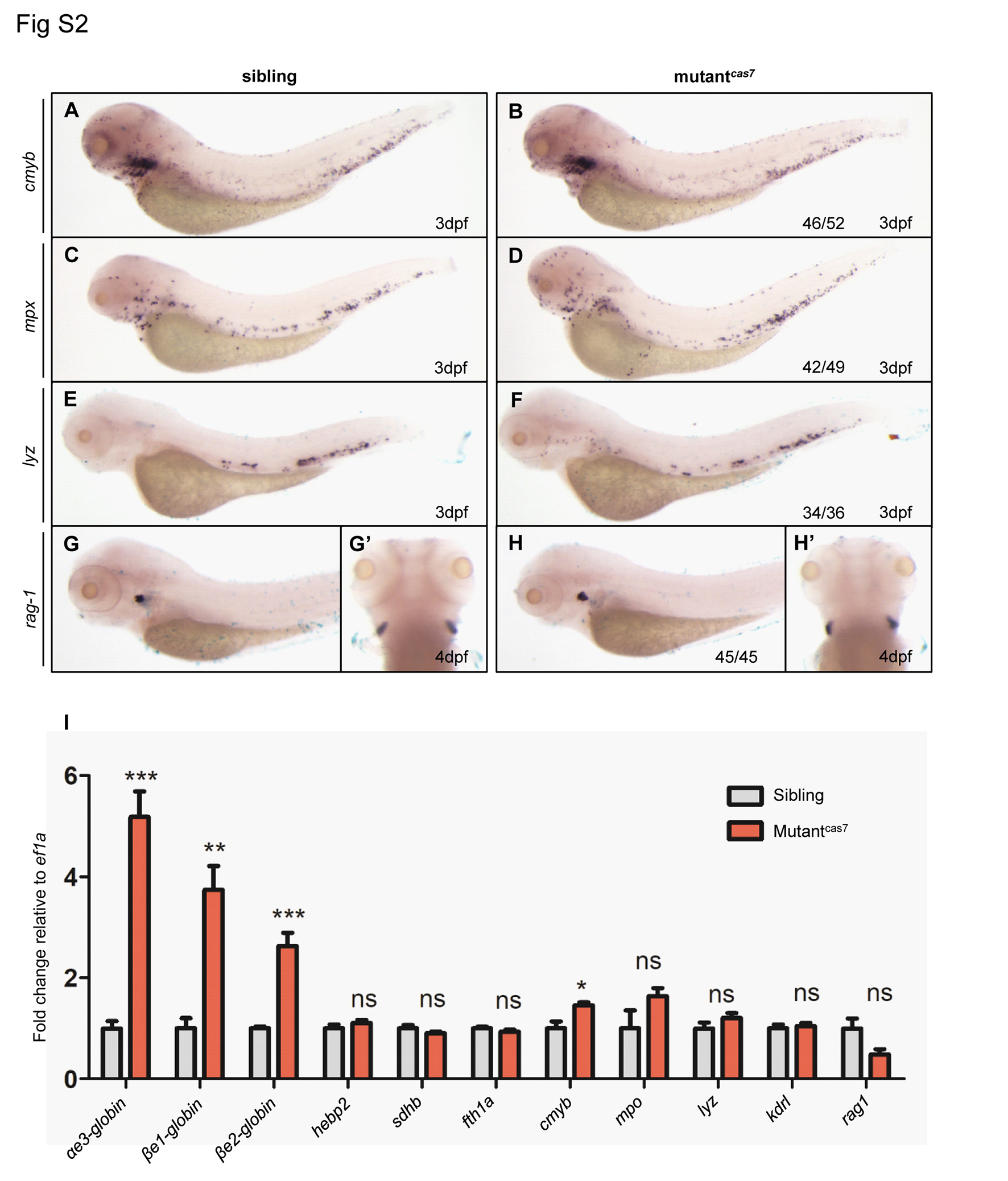

Supplement: Supplementary Figure 2 — WISH analysis of hematopoietic lineages at 3/4 dpf. WISH analysis of cmyb (A,B), mpx (C,D), lyz (E,F), and rag-1 (G,H′) expression shows no significant difference between sibling and mutantcas7 at 3/4 dpf. After WISH and photographing, all embryos were extracted for genomic DNA and genotyped by sequencing, then the mutant percentage was evaluated. The percentages of embryos in a het-het incross clutch with the expression pattern as shown in the mutantcas7 column is listed at the bottom of each panel. (I) The relative expression of αe3-globin, βe1-globin, βe2-globin, hebp2, sdhb, fth1a, cmyb, mpo, lyz, kdrl, and rag1 in sibling and mutantcas7 embryos at 3 dpf. Error bars represent SEM. ns, not significant; *p ≤ 0.05; ∗∗p ≤ 0.01; ∗∗∗p ≤ 0.001. [file Image_2.JPEG]

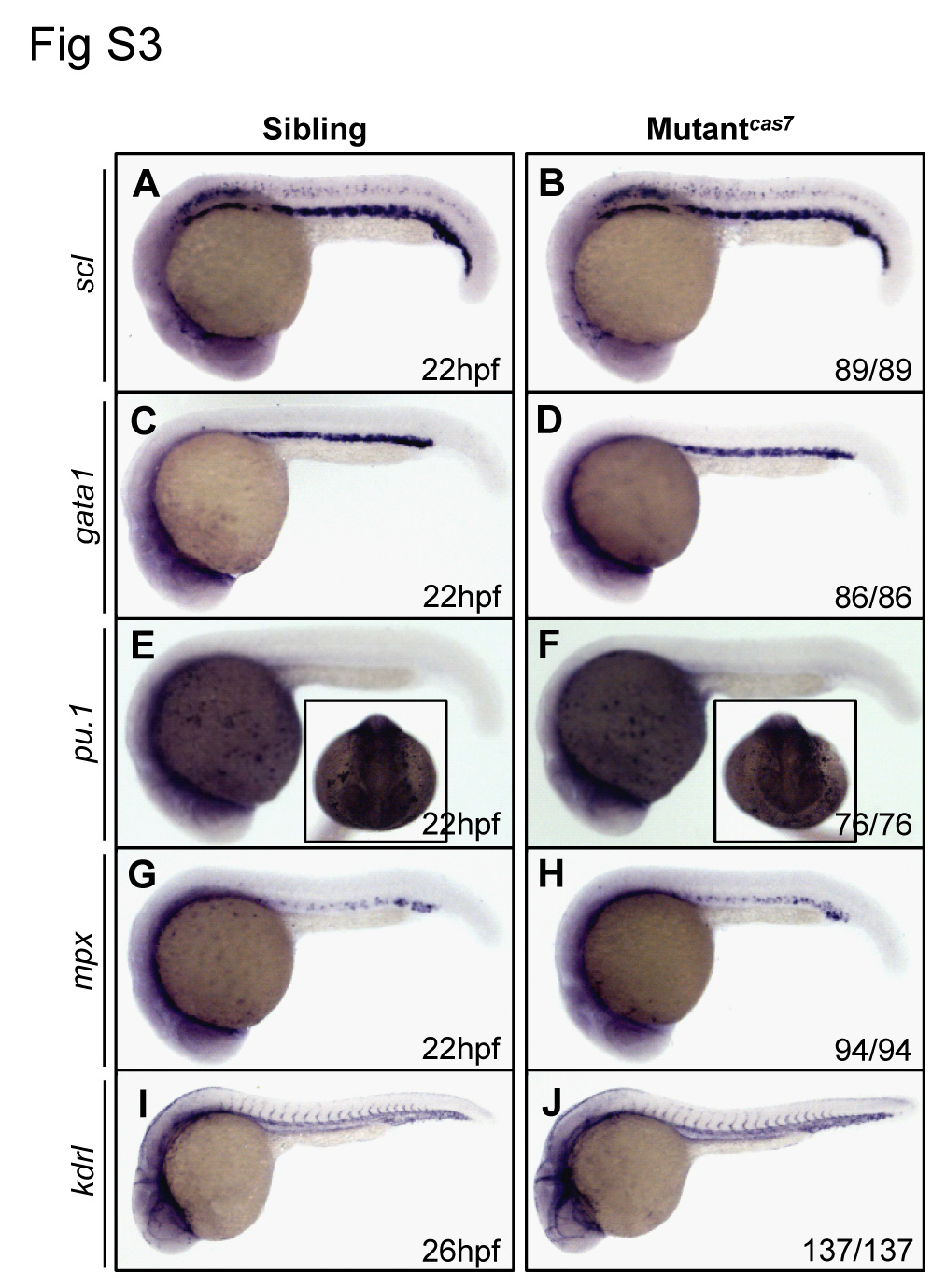

Supplement: Supplementary Figure 3 — Normal primitive hematopoiesis and vascular morphogenesis in mutantcas7. Sibling and mutantcas7 embryos were fixed at 22 or 26 hpf, followed by WISH analysis of scl, gata1, pu.1, mpx, and kdrl expression. Expressions of scl (A,B), gata1 (C,D), pu.1 (E,F), mpx (G,H), and kdrl (I,J) show no significant difference between wild-type siblings and mutants. Insets in panels (E,F) show dorsal views of the head staining region. After WISH and photographing, all embryos were extracted for genomic DNA and genotyped by sequencing, then the mutant percentage was evaluated. The number and percentage of het-het incross embryos with the in situ pattern are listed at the bottom of the mutantcas7 panels. [file Image_3.JPEG]

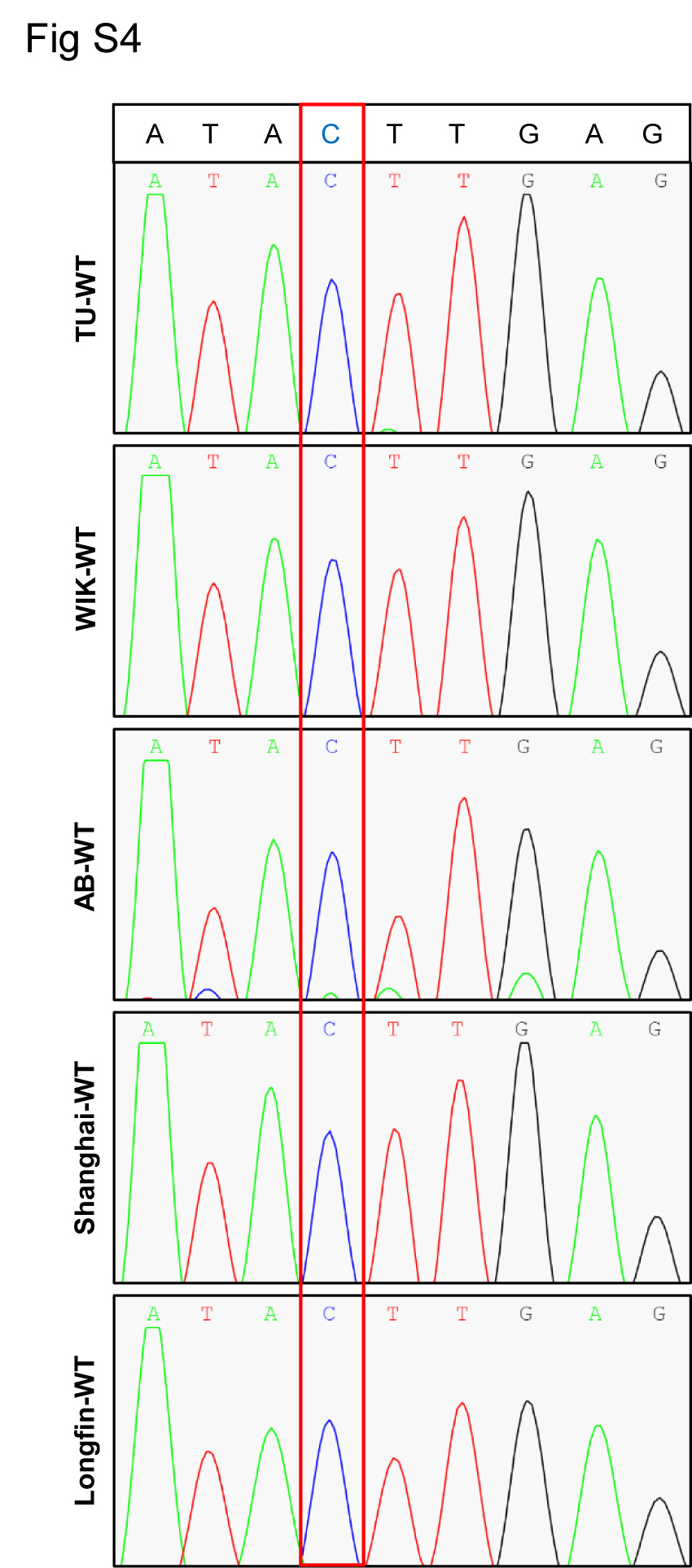

Supplement: Supplementary Figure 4 — The point mutation (C-G) in mutantcas7 was not found in all five wild-type strains, indicating that C-G transition at the site is not a SNP. Sequencing electropherogram of the mutation site of tprb genomic DNA from five wild-type strains. Genomic DNA were extracted each from 20 to 30 embryos of TU, AB, WIK, Longfin, and Shanghai strains, and then sequenced for tprb gene. This point mutation (C-G) in mutantcas7 was not found in all five wild-type strains. [file Image_4.JPEG]

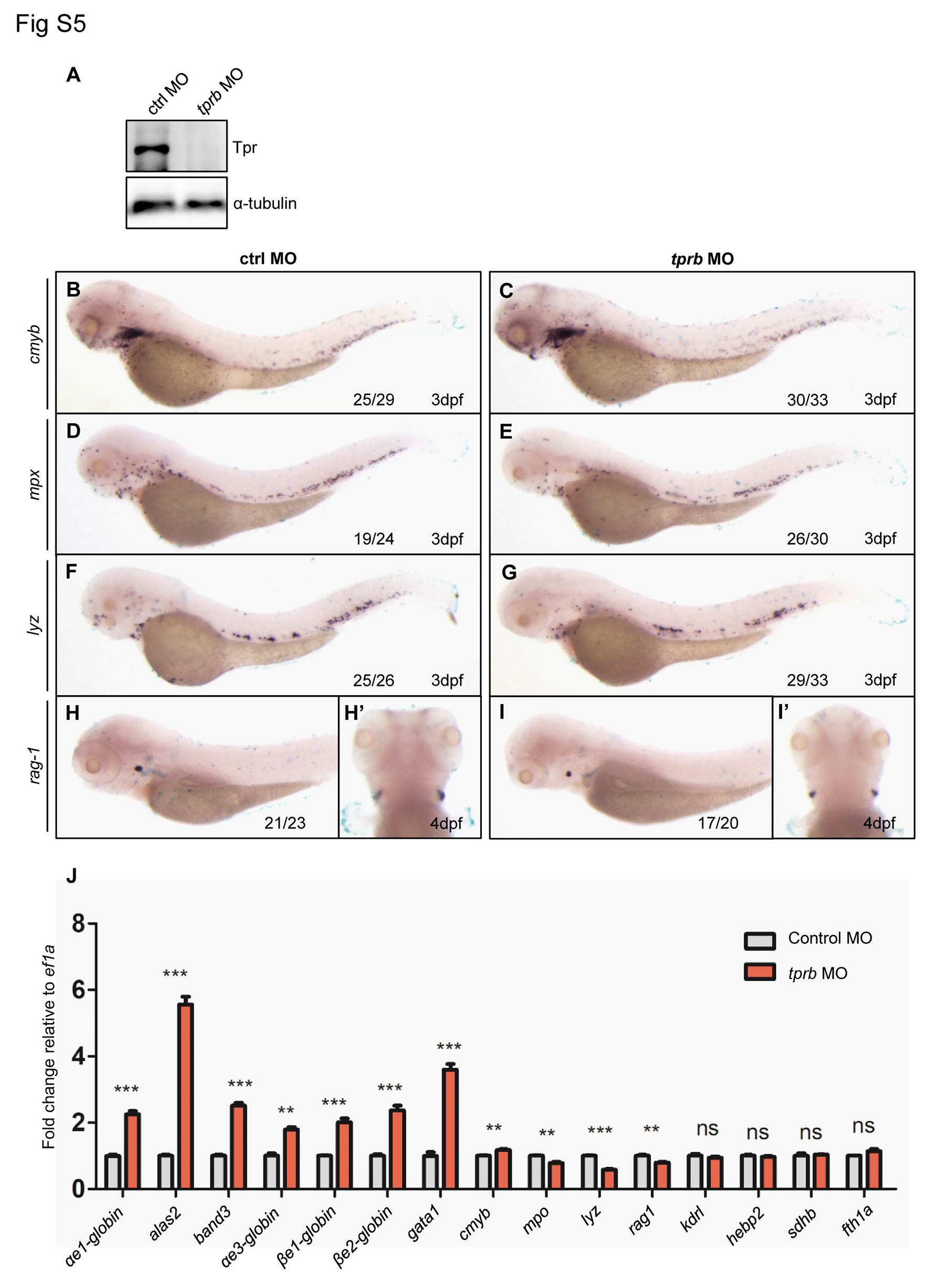

Supplement: Supplementary Figure 5 — WISH analysis of hematopoietic lineages in tprb morphants at 3/4 dpf. (A) Representative immunoblotting images of Tpr in control and tprb morphants. WISH analysis of cmyb (B,C), mpx (D,E), lyz (F,G), and rag-1 (H,I′) expression show no significant difference between control and tprb morphants at 3/4 dpf. After WISH and photographing, all embryos were extracted for genomic DNA and genotyped by sequencing, then the mutant percentage was evaluated. The percentage of indicated phenotype is listed at the bottom of each panel. (J) The relative expression of αe1-globin, alas2, band3, αe3-globin, βe1-globin, βe2-globin, gata1, hebp2, sdhb, fth1a, cmyb, mpo, lyz, kdrl, and rag1 at 3 dpf in tprb morphants. Error bars represent SEM. ns, not significant; ∗∗p ≤ 0.01; ∗∗∗p ≤ 0.001. [file Image_5.JPEG]

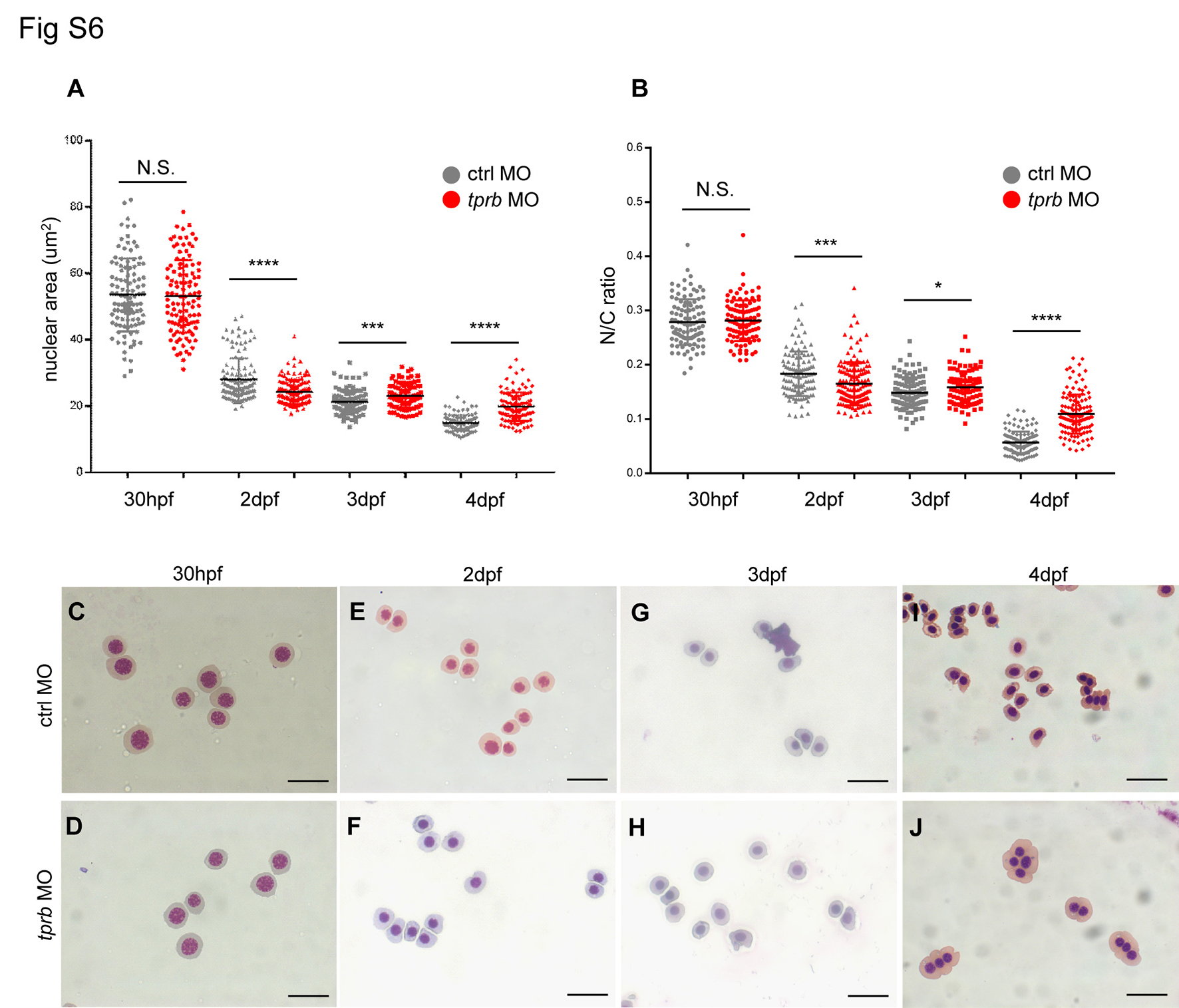

Supplement: Supplementary Figure 6 — Giemsa staining in circulating erythroid cells in control and tprb morphants at different time stages. (A) Quantitative analysis of each erythroid cell nuclear area. (B) Quantitative analysis of nucleus-to-cytoplasm (N/C) ratio. Error bars represent SEM. ns, not significant; ****p ≤ 0.0001; ∗∗p ≤ 0.01; ∗∗∗p ≤ 0.001. (C–G) Representative images of Giemsa staining in circulating erythroid cells at different stages: 30 hpf (C,D), 2 dpf (E,F), 3 dpf (G,H), and 4 dpf (I,J). Scale bars represent 20μm. [file Image_6.JPEG]

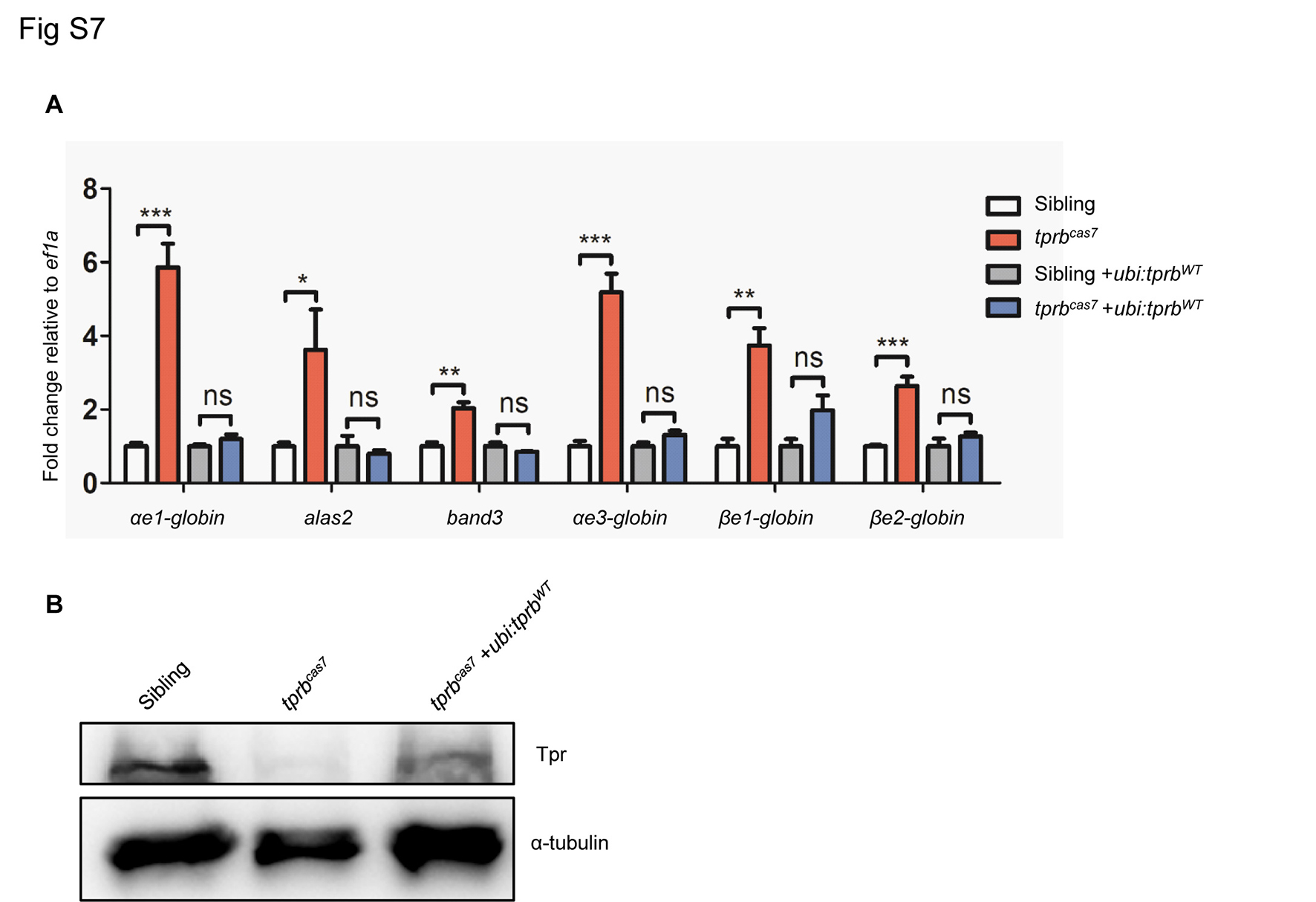

Supplement: Supplementary Figure 7 — The result of tprbcas7 rescue experiment at 4 dpf. Transient expression of wild-type tprb under the ubiquitin promoter in mutantcas7 could rescue the phenotypes. Mutantcas7 embryos at one-cell stage were injected the tol2 plasmid with tprbWT expression driven by ubiquitin promoter. Then embryos with mCherry fluorescence would be selected for downstream analysis. At 4 dpf, every embryo was cut off in head for genotyping, then the trunk and tail of the embryo was taken for RNA or protein extraction. After genotyping the mutant embryos, we pooled three to five of the same phenotype embryo RNA extraction and took the qPCR experiments. (A) The relative expression of αe1-globin, alas2, band3, αe3-globin, βe1-globin, and βe2-globin at 4 dpf. Error bars represent SEM. ∗∗p ≤ 0.01; ∗∗∗p ≤ 0.001. (B) Representative immunoblotting images of Tpr in tprb mutants rescued by tol2 plasmid with tprbWT expression. [file Image_7.JPEG]

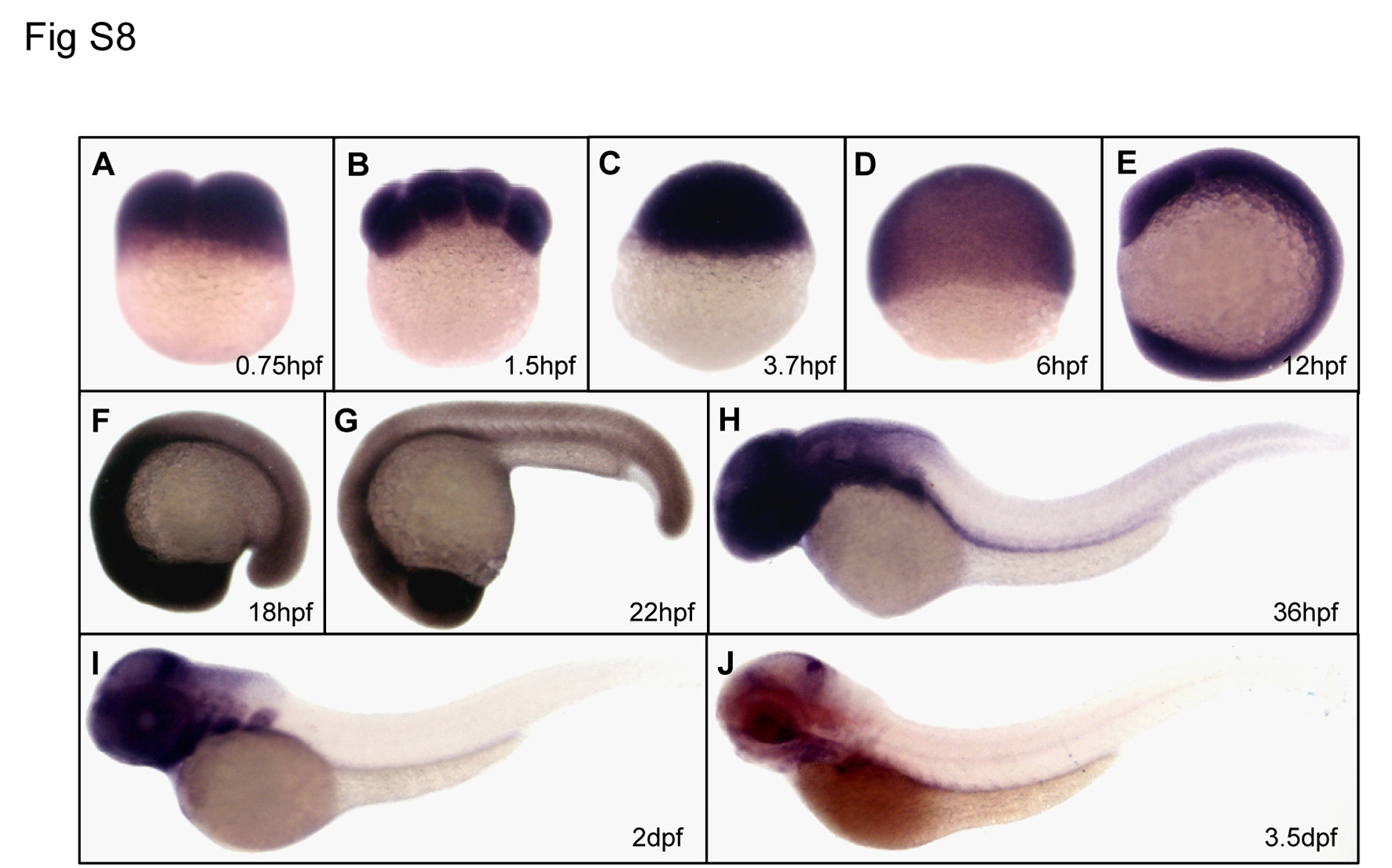

Supplement: Supplementary Figure 8 — Expression pattern of tprb mRNA during zebrafish embryogenesis. (A–J) WT (TU strain) embryos were fixed at the time points as indicated in each panel, followed by WISH analysis of tprb mRNA expression. [file Image_8.JPEG]

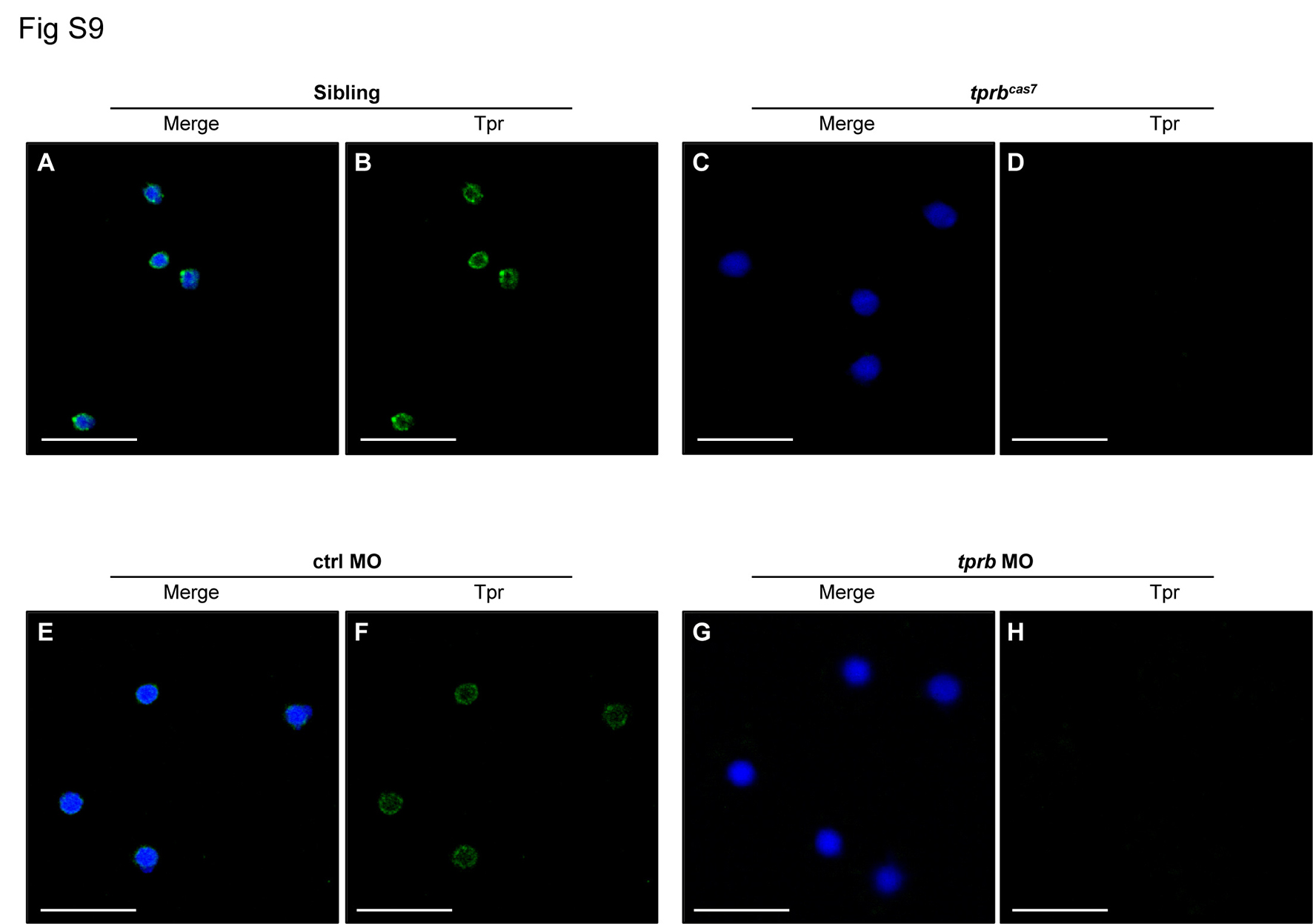

Supplement: Supplementary Figure 9 — The endogenous Tpr protein is located on the nuclear membrane and Tpr protein is missing in tprb mutant or morphant. Representative images of Tpr immunofluorescence in sibling (A,B) and mutants (C,D) at 3 dpf. All embryos were extracted for genomic DNA and genotyped by sequencing. Representative images of Tpr immunofluorescence in control morphants (E,F) and tprb morphants (G,H) at 3 dpf. Scale bars represent 20 μm. [file Image_9.JPEG]

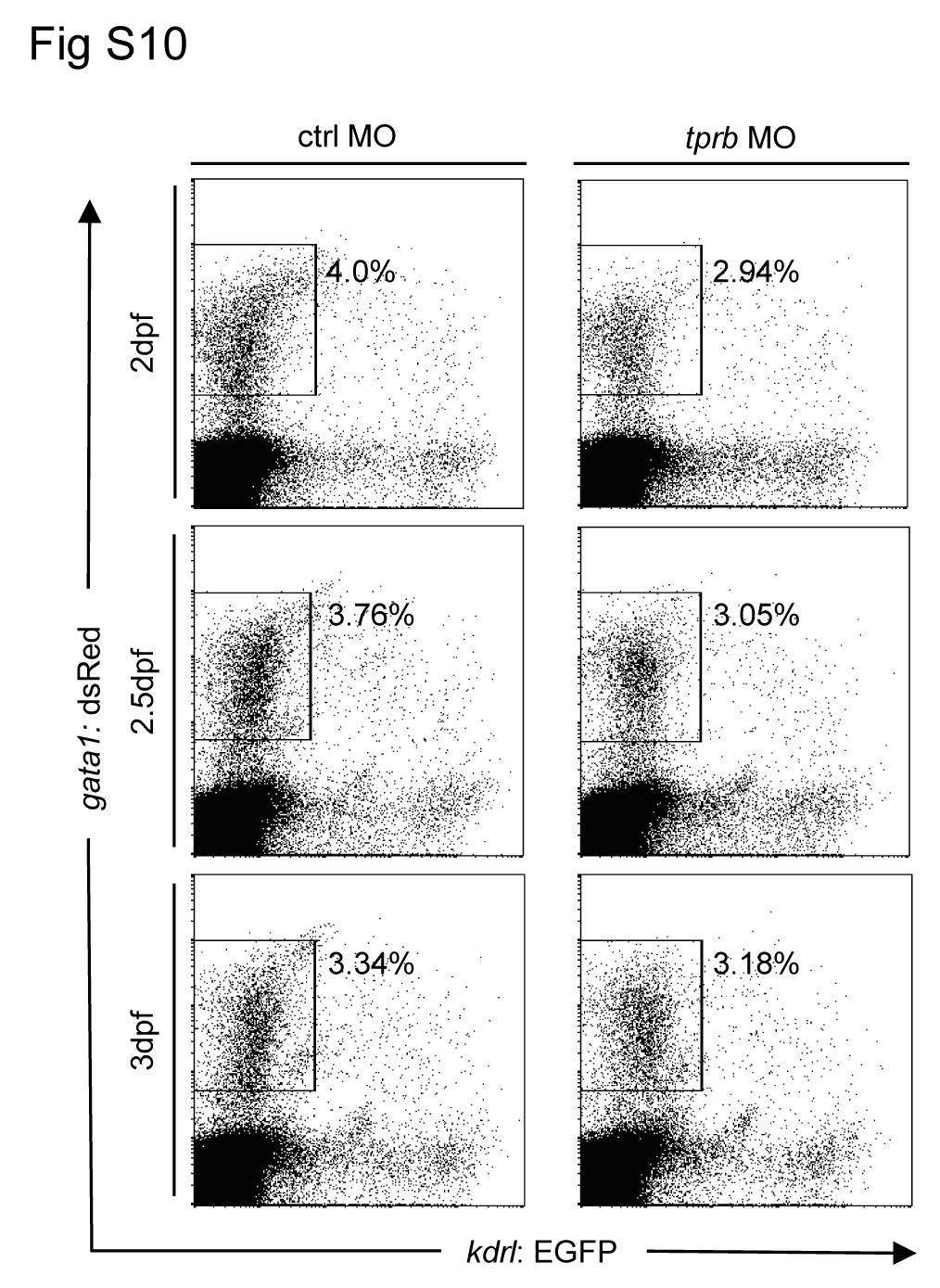

Supplement: Supplementary Figure 10 — Flow cytometry analysis of erythrocytes from control and tprb morphants. Flow cytometry analysis of gata1+ cells from Tg(gata1: DsRed; kdrl: EGFP) transgenic line in control and tprb morphants at different time points. The ratio of gata1+ erythrocytes between control and tprb morphants was similar. [file Image_10.JPEG]

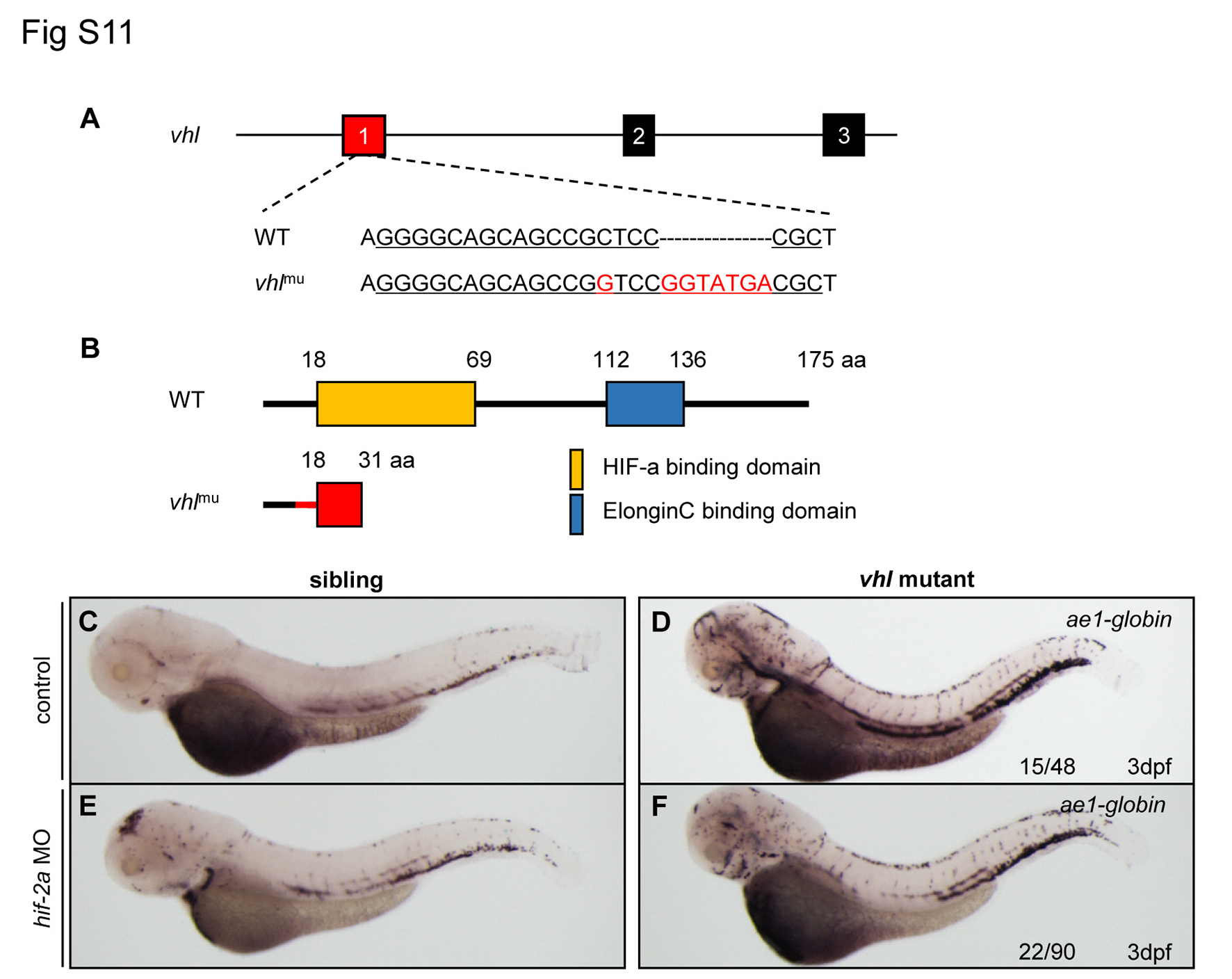

Supplement: Supplementary Figure 11 — Generation and phenotype characterization of zebrafish vhl mutant. (A,B) Generation of vhl mutant via CRISPR-Cas9 technique. The alignment of WT and mutated sequences is listed. The underlined sequence is vhl gRNA target site. The sequencing result of vhl genomic DNA showed changed bases (shown in red) at exon 1 (A), which caused a premature stop codon leading to the production of a truncated 31-amino-acid Vhl protein (B). (C–F) WISH analysis of ae1-globin expression in sibling and vhl mutants at 3 dpf under the control and hif-2a morpholino (E,F) injection, indicating that the hif-2a MO can partially rescue the increased expression of ae1-globin in vhl mutant. [file Image_11.JPEG]

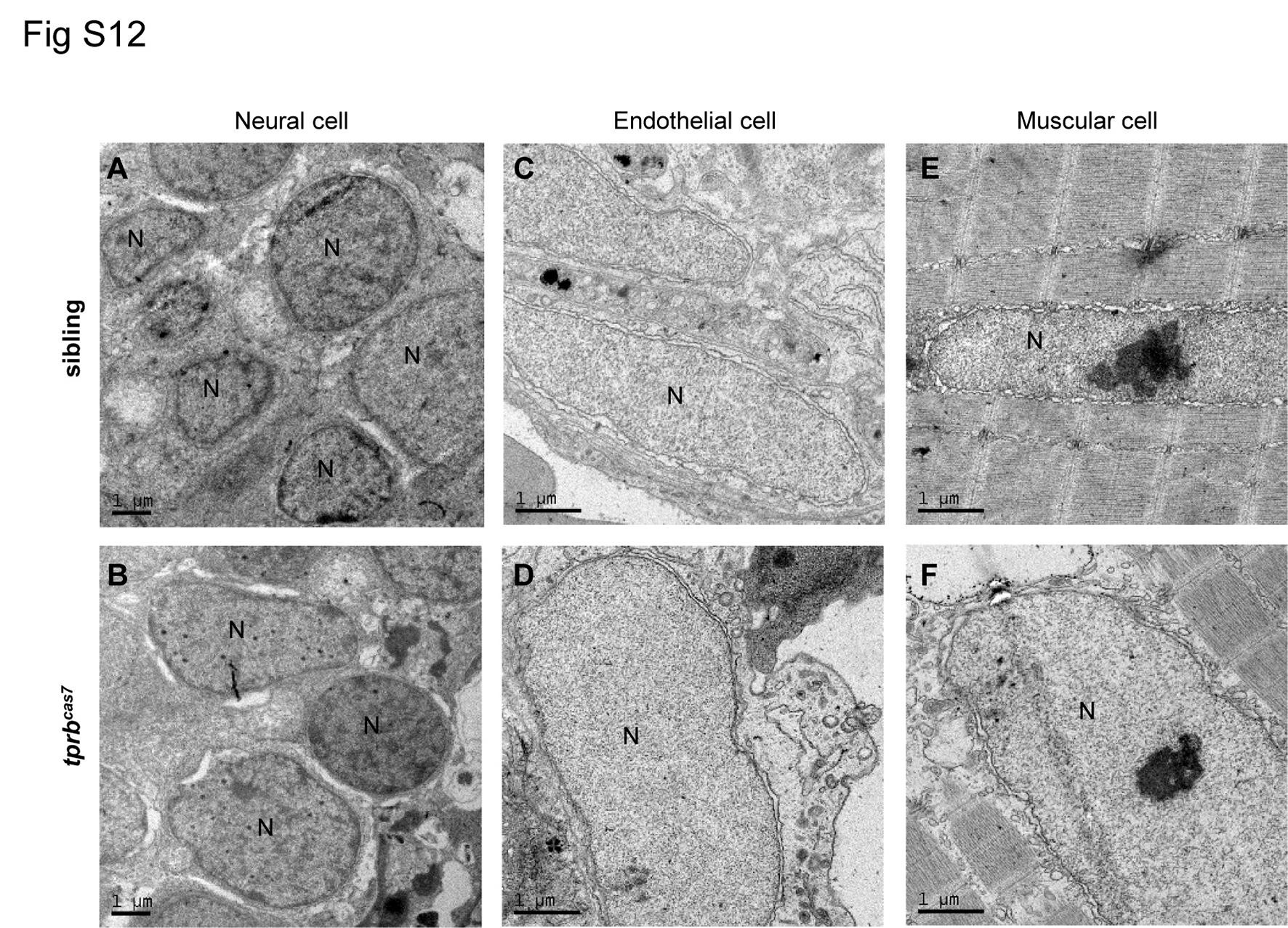

Supplement: Supplementary Figure 12 — The other tissue cells in tprbcas7 mutant have normal chromatin organization. (A–F) Representative TEM images of chromatin organization within the tissue cells show no obvious difference between sibling and tprbcas7 mutants at 4 dpf, including neural cells (A,B), endothelial cells (C,D), and muscular cells (E,F). [file Image_12.JPEG]

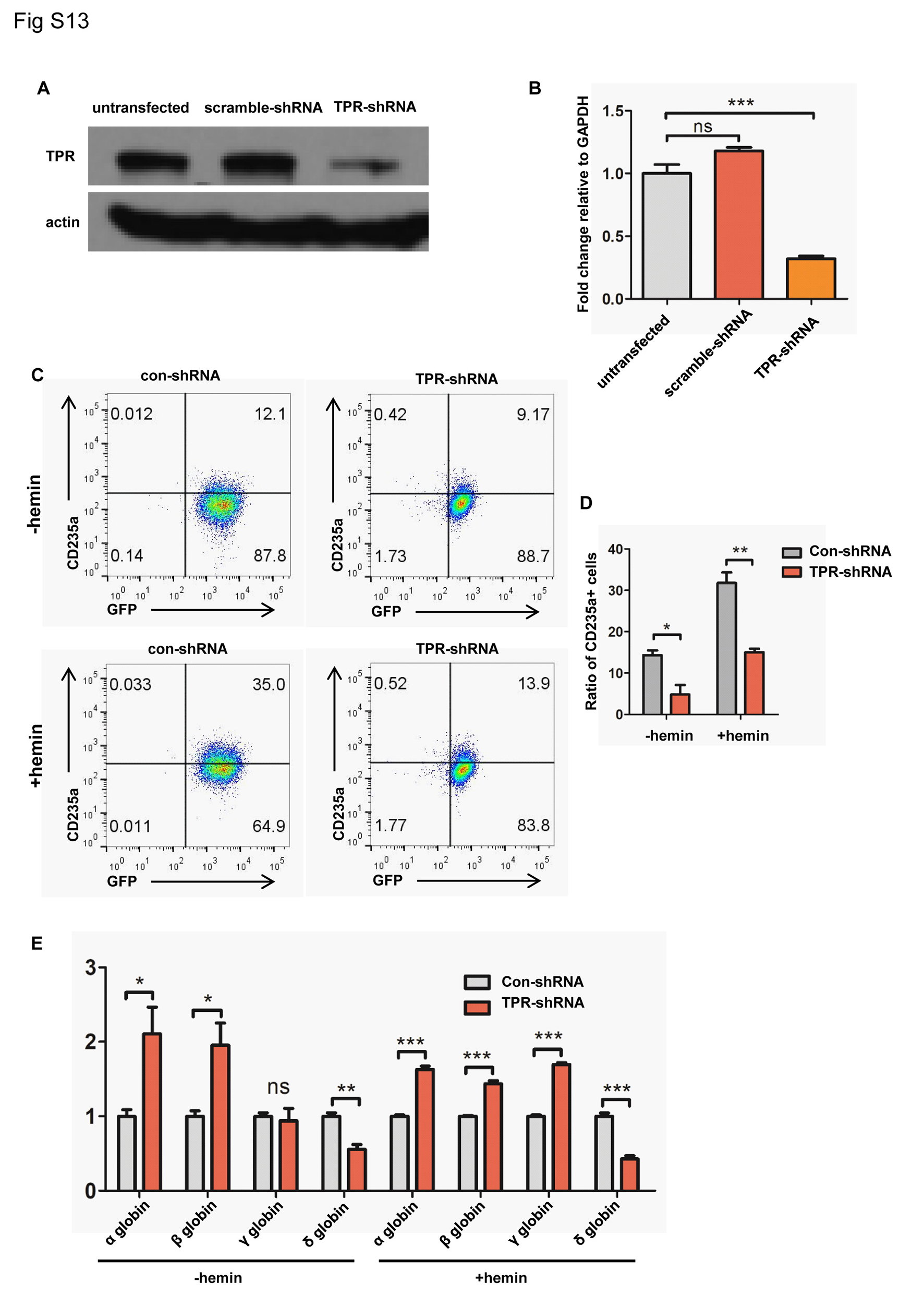

Supplement: Supplementary Figure 13 — TPR is also important for erythroid differentiation in mammalian. (A) Representative immunoblotting images of TPR shRNA and scramble shRNA in K562 cell line. Tubulin serves as the loading control. (B) The relative expression of TPR in different groups. (C) Representative FACS images of the ratio of CD235a-positive cells with or without hemin treatment. (D) Quantitative analysis of panel (C). (E) The relative expression of α/β/γ/δ-globins with or without hemin treatment. Error bars represent SEM. ns, not significant; *p ≤ 0.05; ∗∗p ≤ 0.01; ∗∗∗p ≤ 0.001. [file Image_13.JPEG]

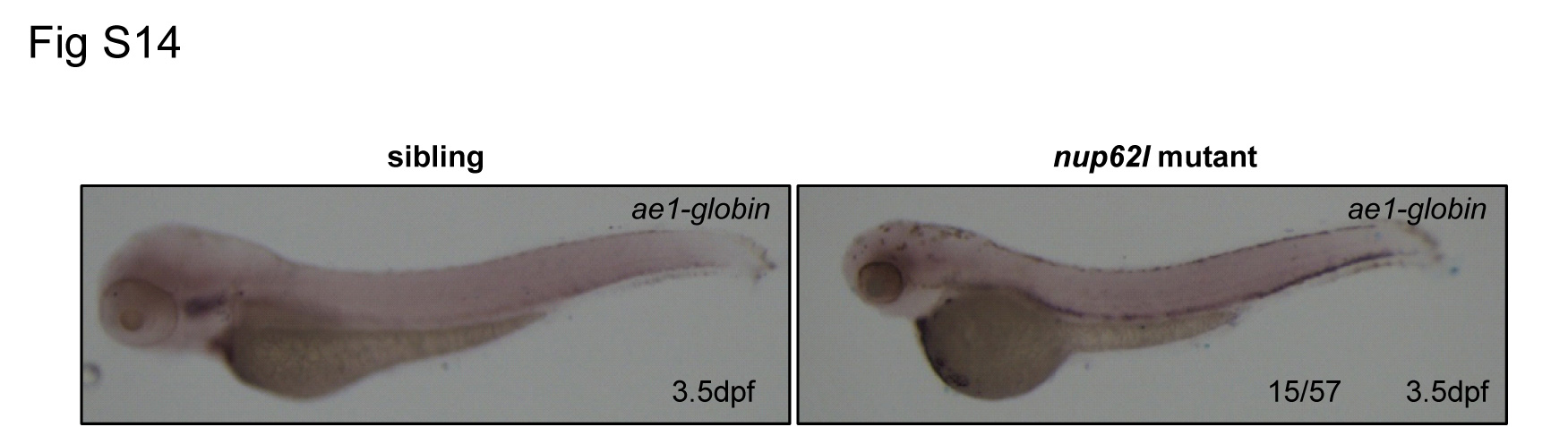

Supplement: Supplementary Figure 14 — Zebrafish nup62l mutants represent similar phenotype with tprbcas7 mutants. (A,B) WISH analysis of ae1-globin show the increased expression in nup62l mutants compared with sibling at 3.5 dpf. The number and percentage of het-het incross embryos with the in situ pattern in a clutch are listed at the bottom of the mutant panels. [file Image_14.JPEG]
