# Supplementary material for: Tpr Deficiency Disrupts Erythroid Maturation With Impaired Chromatin Condensation in Zebrafish Embryogenesis
Source: Front Cell Dev Biol. 2021 Oct 13;9:709923. doi: 10.3389/fcell.2021.709923 (PMC8548687; doi:10.3389/fcell.2021.709923)
Supplement: Supplementary file 15 [file Data_Sheet_1.PDF]

**Supplementary information.** Summarized information of oligos and primers used in this study

**Table S1**. Primers used in positional cloning

| name | sequence | notes |
|---|---|---|
| z45247-F | ACTGTTTCCCTAATGAAAAACATT | for positional cloning (SSLP markers) |
| z45247-R | GGCGAGAGTTGAGTTTGAGG | |
| 297-01-F | GCGACAGTGTGAAACGTAGG | |
| 297-01-R | AGCATGCGCTGATACAGAAA | |
| 294-04-F | GTAACTTCCGATGGCACGTC | |
| 294-04-R | CACCTGCTTATGGGAGGAGA | |
| SNP1-F | GCCTTACTGGATGGTCAGGA | |
| SNP1-R | AGACAGTTGCTTGGTGCAAA | |
| SNP2-F | TGGGTGCTTGAAAAGTGCTA | |
| SNP2-R | GCAATGTCAGTTTGGAGTGTAA | |
| SNP3-F | TGGACAAAACCTTGATCGAC | |
| SNP3-R | ACACTCAATAATGGCCAAAAA | |
| SNP4-F | GGCTTATGAAACATACGCACAA | |
| SNP4-R | AACAGTTGGGTTGACATTTCG | |
| *tprb* -seq-F | AAGGACGCAGTTTTCACGTT | for sequencing of *tprb* genomic DNA (*cas7* mutant) |
| *tprb* -seq-R | GCAGAACAACACGTCCAAGA | |

**Table S2**. Morpholinos used in this study

| name | sequence | notes |
|---|---|---|
| control MO | GGTCAGCATTCAAGAGACCATGCAT | |
| *tprb* ATG MO | GTATCTGCTGCAACACAGCCGCCAT | |
| *hif-2a* ATG MO | CGCTGTTCTCGCGTAATTCCCGCAG | |

**Table S3**. Oligos and Primers used in CRISPR–Cas9 mutagenesis

| name | sequence | notes |
|---|---|---|
| *tprb* gRNA target site | GTCACCTGATCTTGAAGG | |
| *vhl* gRNA target site | GGGGCAGCAGCCGCTCCCGC | |
| tracr rev | AAAAAAAGCACCGACTCGGTGCCAC | reverse primer for gRNA oligo |
| *tprb* -PCR-F | GTGACCTGGCTGAACACTGA | for sequencing of *tprb* genomic DNA (*tprb* $^{-3+1}$ mutant) |
| *tprb* -PCR-R | GTGTGCGAAGACCCTAAAGC | |
| *vhl* PCR-F | AGCACGGACGTATGTGTGAC | for sequencing of *vhl* genomic DNA (*vhl* mutant) |
| *vhl* PCR-R | CTCCGAGGAAGTTGATCCAG | |

**Table S4**. Primers used in plasmid construction

| name | sequence | notes |
|---|---|---|

| | | |
|---|---|---|
| tpr17941-1-F | AGCACGTTTGCATTTGACG | for sequencing of *tprb* cDNA |
| tpr17941-1-R | CGGGACAGAGACAGCAGTT | |
| tpr17941-2-F | AACGAATGGAGCAGGAGAAA | |
| tpr17941-2-R | AATGCTCTTGGGTCTCAACG | |
| tpr17941-3-F | GACCATAATGTCCCCCACTG | |
| tpr17941-3-R | TGCCACAGTGTCAACCTTCT | |
| tpr17941-4-F | GAAACGCAGTGAAGTGGAAA | |
| tpr17941-4-R | GGACTCTTTCTCCTGGGTCA | |
| tpr17941-5-F | CCTGCGTAAAGAGAGGGACA | |
| tpr17941-5-R | CCTTGTCCATTTCCAGCAGT | |
| tpr17941-6-F | GCAGGTAACTGAGCAAGTTCG | |
| tpr17941-6-R | CTCTCTGCGCACAAATCTGA | |
| tpr17941-7-F | AGCCACCACTGAGAGTCCAC | |
| tpr17941-7-R | TCCGAGGTTCTCCCTTAGGT | |
| tpr17941-8-F | GCCGACTAAAAGCTGATGCT | |
| tpr17941-8-R | GGTCTCGTAGCTCCCTTTCC | |
| tpr17941-9-F | GAACGAGGAGCTGAAGCAAC | |
| tpr17941-9-R | GCACTACCAGGAGATGCAGA | |
| tpr17941-10-F | GCTCTAATGCTGCCAACCTC | |
| tpr17941-10-R | AGGGCCCTCTGTGTCATCT | |
| tpr17941-11-F | GGGGAAGAGGGAGATGAAAG | |
| tpr17941-11-R | TCACTCGCTTCAGATACAGCA | |
| tpr17941-12-F | GGACGCAGTGTACCCACAAC | |
| tpr17941-12-R | GCATACAACTAACAAACGACAGC | |
| *tprb* -BamHI-F | CTACGGATCCAATGGCGGCTGTGTTGCAGC | for *tprb* $^{WT}$ cDNA cloning into pCS2+ plasmid |
| *tprb* -EcoRI-R | AGCAGAATTCGATGAAGACTAGAATGAAGA | |
| *tprb* $^{WT}$-BamHI-F | GTTGGGATCCAATGGCTGCGGTACTACAACAAATCCTTGA | for construction of pUbi-*tprb* $^{WT}$-P2A-mCherry-Tol2 plasmid |
| *tprb* $^{WT}$-BamHI-R | GTGTGGATCCAATCTGGCAAAGCGTCCTCTCACACCCATA | |
| *tprb* -probe-F | ATTACTCGAGGTCTTGGAGCGACAGACTCC | for generation of *tprb* WISH probe |
| *tprb* -probe-R | CACATCTAGAGATGATGGTTCCTGGCTTGT | |

**Table S5**. Primers used in quantitative PCR

| name | sequence | notes |
|---|---|---|
| *ef1a* QPCR-F | TTGAGAAGAAAATCGGTGGTGCTG | |
| *ef1a* QPCR-R | GGAACGGTGTGATTGAGGGAAATTC | |
| *epo* QPCR-F | AGGAGGCAGGATATGGACTATTAC | |
| *epo* QPCR-R | ACAGTTGGAGGTGCTTGAGG | |
| *vegfaa* QPCR-F | ACACACCAAGTGTGAATGCAG | |

| | | |
|---|---|---|
| *vegfaa* QPCR-R | GCAAGGCTCACAGTGGTTTT | for zebrafish QPCR |
| *ae1-globin* QPCR-F | CCAGGATGTTGATTGTCTAC | |
| *ae1-globin* QPCR-R | CAGTCTTGCCGTGTTTC | |
| *be1-globin* QPCR-F | CTTGACCATCGTTGTTG | |
| *be1-globin* QPCR-R | GATGAATTTCTGGAAAGC | |
| *alas2* QPCR-F | AAAAGCTGCTCAATCCTCTGA | |
| *alas2* QPCR-R | TGCCGCTTTGGCTCTTTAT | |
| *ae3-globin* QPCR-F | GCAAAGGACAAAGCGAACGT | |
| *ae3-globin* QPCR-R | AGGAGAGTTGGGGCTTAGGT | |
| *be2-globin* QPCR-F | GATCCTGACAACTTCAGGCTGCT | |
| *be2-globin* QPCR-R | TGCGTTAGAGAGCAAACATACAATG | |
| *hebp2* QPCR-F | ACGACACCGGTGAGTTGTTT | |
| *hebp2* QPCR-R | CCACCAAAGGTCCTGACGAA | |
| *sdhb* QPCR-F | CTGTGGCTCGTGTGCTATGA | |
| *sdhb* QPCR-R | TAGAAGTTGCTCATGTCGGGC | |
| *fth1a* QPCR-F | TGCAGGACGTGAAGAAACCAG | |
| *fth1a* QPCR-R | TGTGAGGGTCGTTGTGTTGA | |
| *cmyb* QPCR-F | TCGATGCTATTGATTCGGCCC | |
| *cmyb* QPCR-R | TTGGGCGTTGAGCAAAGAAA | |
| *mpo* QPCR-F | AATCTGCGACAACACCGGAA | |
| *mpo* QPCR-R | ATGCCACCATCACCAGTCTC | |
| *lyz* QPCR-F | GTGGCCTGTTCAGACTTGCT | |
| *lyz* QPCR-R | CGCTGCTCACAGCCTTTTAC | |
| *kdrl* QPCR-F | AGGACCCAGACTATGTCCGC | |
| *kdrl* QPCR-R | GGTAAGGGGAGGCACCAAGA | |
| *rag1* QPCR-F | CTGGCATTGAGAGCTGGGAA | |
| *rag1* QPCR-R | TGTGCAGGGGCTGGAATATC | |
| *band3* QPCR-F | ATTGGACCCTCAAGTGCTGT | |
| *band3* QPCR-R | CCATCATGTGCTCCGTCTTA | |
| *gata1a* QPCR-F | AACGACATCTTCAATACTACACTTGC | |
| *gata1a* QPCR-R | GGACACCCAACGAGAAGG | |
| *GAPDH* QPCR-F | CCCCGGTTTCTATAAATTGAGC | |
| *GAPDH* QPCR-R | CTTCCCCATGGTGTCTGAG | |
| *TPR* QPCR-F | TTCACGACTTCGTCAGGATCT | |
| *TPR* QPCR-R | TTCCTTTTCAGTTATCTGTTGTCG | |
| *VHL* QPCR-F | ATGGCTCAACTTCGACGGC | |
| *VHL* QPCR-R | CCAGAAGCCCATCGTGTGTC | |

| | | |
|---|---|---|
| *PDK1* QPCR-F | AGGGATGTGAATGGGCAGT | for human cells QPCR |
| *PDK1* QPCR-R | CTAAGCAGCTCTGGGCAAA | |
| *PGK1* QPCR-F | ACGGATCAGATGTCTATATTGCTG | |
| *PGK1* QPCR-R | TTATCTAATTGTCCCATCTCTCCA | |
| *EPO* QPCR-F | TGTTTTCGCACCTACCATCA | |
| *EPO* QPCR-R | AAGTCACAGCTTGCCACCTAA | |
| *VEGF QPCR-F* | GGGCAAATATGACCCAGTTTT | |
| *VEGF QPCR-R* | TGTCTTTCTGTCCGTCTGACC | |
| α *globin QPCR-F* | TGGTCCCCACAGACTCAGAGA | |
| α *globin QPCR-R* | CGGCCTTGACGTTGGTCTT | |
| *β globin QPCR-F* | GAAGGCTCATGGCAAGAAAG | |
| β *globin QPCR-R* | CACTGGTGGGGTGAATTCTT | |
| *γ globin QPCR-F* | CCAGCTGAGTGAACTGCACTGT | |
| γ *globin QPCR-R* | ACGGTCACCAGCACATTTCC | |
| *δ globin QPCR-F* | CCTAAGGTGAAGGCTCATGG | |
| *δ globin QPCR-R* | AGCACACACACCAGCACATT | |

**Table S6**. Oligos used in siRNA/shRNA transfection

| name | sequence | notes |
|---|---|---|
| TPR siRNA-1F | GCCAUUCAGAGCCAAUUUATT | |
| TPR siRNA-1R | UAAAUUGGCUCUGAAUGGCTT | |
| TPR siRNA-2F | GCCUCAAGAACCUUCUAAUTT | |
| TPR siRNA-2R | AUUAGAAGGUUCUUGAGGCTT | |
| Negative control-F | UUCUCCGAACGUGUCACGUTT | |
| Negative control-R | ACGUGACACGUUCGGAGAATT | |
| VHL siRNA-F | ACACAGGAGCGCAUUGCACAU | |
| VHL siRNA-R | AUGUGCAAUGCGCUCCUGUGU | |
| TPR shRNA | GGTGGAGAGCGAACAACAG | |

**Table S7**. Primers used in quantitative ChIP

| name | sequence | notes |
|---|---|---|
| *ae1-globin* ChIP-PCR-F | CTCAGAGGTCCCCCTGCT | |
| *ae1-globin* ChIP-PCR-R | TGTGGAAGGATGACTTTACAGC | |
| *be1-globin* ChIP-PCR-F | TCATCGTGTACCCCTGGACT | |
| *be1-globin* ChIP-PCR-R | GCAGCAACCATTGGGTTT | |
